# Nutrition Interventions for Children with Cerebral Palsy in Low- and Middle-Income Countries: A Scoping Review

**DOI:** 10.3390/nu14061211

**Published:** 2022-03-12

**Authors:** Israt Jahan, Risad Sultana, Mohammad Muhit, Delwar Akbar, Tasneem Karim, Mahmudul Hassan Al Imam, Manik Chandra Das, Hayley Smithers-Sheedy, Sarah McIntyre, Nadia Badawi, Gulam Khandaker

**Affiliations:** 1 CSF Global, Dhaka 1213, Bangladesh; arda.jahan89@gmail.com (I.J.); sultana.risad@gmail.com (R.S.); mmuhit@hotmail.com (M.M.); tasneem.karim.tk@gmail.com (T.K.); physiomahmud@yahoo.com (M.H.A.I.); manikchandradas10@gmail.com (M.C.D.); 2 Asian Institute of Disability and Development (AIDD), University of South Asia, Dhaka 1212, Bangladesh; 3 School of Health, Medical and Applied Sciences, Central Queensland University, Rockhampton, QLD 4701, Australia; 4 School of Business and Law, Central Queensland University, Rockhampton, QLD 4701, Australia; d.akbar@cqu.edu.au; 5 Specialty of Child and Adolescent Health, Sydney Medical School, The University of Sydney, Sydney, NSW 2145, Australia; 6 Cerebral Palsy Alliance, Sydney Medical School, The University of Sydney, Camperdown, NSW 2006, Australia; hsmitherssheedy@cerebralpalsy.org.au (H.S.-S.); smcintyre@cerebralpalsy.org.au (S.M.); nadia.badawi@health.nsw.gov.au (N.B.); 7 Central Queensland Public Health Unit, Central Queensland Hospital and Health Service, Rockhampton, QLD 4700, Australia; 8 Grace Centre for Newborn Intensive Care, The Children’s Hospital at Westmead, Sydney Children’s Hospital Network, Westmead, NSW 2145, Australia

**Keywords:** nutrition, intervention, children, cerebral palsy, disability, LMICs

## Abstract

Background: Malnutrition is substantially higher among children with cerebral palsy (CP) in low- and middle-income countries (LMICs) when compared with the general population. Access to appropriate interventions is crucial for better management of malnutrition and nutritional outcomes of those children. We aimed to review the existing evidence on nutrition interventions for children with CP in LMICs. Methods: Online databases, i.e., PubMed and Scopus, and Google Scholar were searched up to 10 January 2022, to identify peer-reviewed publications/evidence on LMIC focused nutritional management guidelines/interventions. Following title screening and abstract review, full articles that met the inclusion/exclusion criteria were retained for data charting. Information about the study characteristics, nutrition interventions, and their effectiveness were extracted. Descriptive data were reported. Results: Eight articles published between 2008 and 2019 were included with data from a total of *n* = 252 children with CP (age range: 1 y 0 m–18 y 7 m, 42% female). Five studies followed experimental design; *n* = 6 were conducted in hospital/clinic/center-based settings. Four studies focused on parental/caregiver training; *n* = 2 studies had surgical interventions (i.e., gastrostomy) and *n* = 1 provided neurodevelopmental therapy feeding intervention. Dietary modification as an intervention (or component) was reported in *n* = 5 studies and had better effect on the nutritional outcomes of children with CP compared to interventions focused on feeding skills or other behavioral modifications. Surgical interventions improved nutritional outcomes in both studies; however, none documented any adverse consequences of the surgical interventions. Conclusion: There is a substantial knowledge gap on nutrition interventions for children with CP in LMICs. This hinders the development of best practice guidelines for the nutritional management of children with CP in those settings. Findings suggest interventions directly related to growth/feeding of children had a better outcome than behavioral interventions. This should be considered in planning of nutrition-focused intervention or comprehensive services for children with CP in LMICs.

## 1. Introduction

Undernutrition is a major global public health challenge, and children with disability, such as cerebral palsy (CP), often suffer from undernutrition, especially in low- and middle-income countries (LMICs). The proportion of malnutrition is substantially higher among children with CP in LMICs compared to the general population [1,2,3,4,5]. Ensuring availability and accessibility to evidence-based nutrition interventions is essential to avert adverse outcomes of malnutrition among those vulnerable children [6].

Nutritional management of children with CP is complex as several interlinked factors interfere with their growth directly and indirectly [7]. While a few factors have been identified as common predictors of malnutrition (e.g., gross motor and oromotor function limitations) [1,8,9], the conceptual framework of malnutrition in children with CP is not yet clearly understood, especially in LMICs. To date, several clinical nutrition guidelines based on current evidence on surgical and non-surgical intervention outcomes have been published [6,10]. However, most of those studies were conducted in high-income countries (HICs) [6], whereas 85% of children with disabilities live in LMICs and the majority have no or limited access to any rehabilitation services [11,12]. With the extreme shortage of trained professionals including dietitians, in addition to the limited availability and accessibility to institutionalized intervention programs in LMICs [13], it is likely that not all the interventions found effective in HIC settings may be applicable in LMIC settings. In absence of optimal management, these children are at high risk of malnutrition, which can in turn impact their functional outcomes, quality of life, and survival [14,15,16].

Any available evidence in the context of LMICs could guide resource mobilization, assist in understanding the need and cost-effectiveness as well as to identify interventions that may improve the nutritional status of children with CP in low-resource settings. This is important to establish a platform for developing and implementing best practice CP-specific nutrition intervention guidelines in LMICs. In this scoping review, we aimed to systematically map the existing evidence on nutritional interventions for children with CP living in LMIC settings. The following research questions were explored: (i) What is known about the available nutrition interventions for children with CP in LMICs? (ii) What are the outcomes of those interventions on the nutritional status of participating children with CP in LMICs?

## 2. Materials and Methods

### 2.1. Study Design

We conducted a scoping review to summarize the available evidence and provide an overview of different intervention programs and their outcomes related to the nutritional status of children with CP in LMICs. A protocol was developed following the Preferred Reporting Items for Systematic Reviews and Meta-analysis (PRISMA) extension for scoping reviews guideline [17] and is available upon request from the corresponding author.

### 2.2. Database Searching

The key words and search strategy were developed by two of the authors (I.J. and G.K.) and pretested prior to the initiation of the search. PubMed (up to 15 December 2021) and Scopus (up to 10 January 2022) were searched to identify relevant articles. Google Scholar was searched for any additional relevant articles or scientific guidelines. The search was not restricted to any specific language. The key terms used included “cerebral palsy”/”neurodevelopmental disorder”; children/adolescent/infant; parent/mother/caregiver; intervention/trial/outcome; training/education/feeding/technique/surgical/teaching; nutrition/malnutrition/growth/health; “low- and middle income countries (LMICs)”, and individual country listed as LMICs according to the 2020 World Bank country classifications by income level (i.e., low-income, lower middle-income, and upper-middle income countries) [18]. The electronic search strategy for Scopus is outlined in Supplementary Table S1. Two reviewers (I.J. and R.S.) independently searched the databases and included articles following the inclusion and exclusion criteria. Any disagreement was resolved by discussion between the reviewers and in consultation with a third reviewer (G.K.).

### 2.3. Inclusion and Exclusion Criteria

Articles that met the following criteria were included in the review: (i) the study participants were children with CP, (ii) the outcome measures included nutritional status as determined by anthropometric measurements, e.g., weight, height/length of children with CP and/or body composition, and (iii) followed analytical study design (e.g., experimental/quasi-experimental, pre- and post-intervention study) or descriptive study with a control group or comparison group (e.g., children with gastrostomy versus children fed orally). Articles were excluded (i) if the study was conducted in HICs, (ii) if data about children with CP could not be differentiated and was reported together with children with other form of impairments, and (iii) if they were not peer-reviewed publications and were protocols, guidelines, book chapters, conference presentations, forewords, or replies to commentaries.

### 2.4. Data Charting Process

Data charting was primarily completed by two independent reviewers (I.J. and R.S.) using an a priori template developed by I.J. and G.K. Information on study design, settings, country, study participants (age, sex, motor function severity, sample size), intervention provided (type, settings, intervention contents, number of sessions as reported, follow-up period), and outcome (measures used and outcome reported) were extracted as available. Any missing information was documented as “not reported”.

### 2.5. Assessment of Risk of Bias and Synthesis of Results

The risk of bias of the included studies were not assessed. Descriptive findings from individual studies including study characteristics (name and economic classification of the country according to the World Bank definitions in 2020, study locations, settings, study design, and study period), participants (sample size, age, sex, gross motor function classification system (GMFCS) level [19]), and outcome measures were reported. The different types of interventions were reported under broad headings, e.g., “training to parents/caregivers”, “gastrostomy tube placement/feeding/nasogastric tube feeding”, “nutritional rehabilitation/therapy”, and “dietary modification”. No statistical test was used considering the study objectives.

### 2.6. Ethics

This study did not require ethics approval as data were collected from existing publications (i.e., secondary data) and no humans were directly contacted to collect/gather any information.

## 3. Results

**Figure 1:**
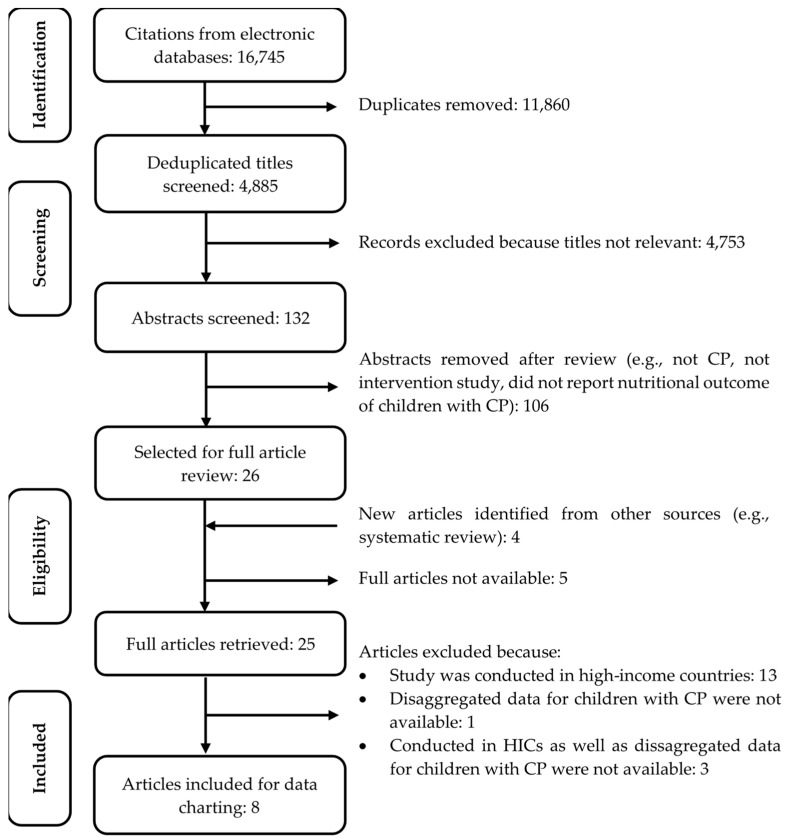
Flow diagram of study selection procedure; CP, Cerebral Palsy.

A total of *n* = 4885 citations were identified from the databases after deduplication. Following title screening, *n* = 132 abstracts were reviewed; of those, *n* = 26 articles were selected for full review. However, of those, *n* = 5 full articles were not available and an additional *n* = 4 were identified from handsearching the bibliographies of selected articles. So, a total of *n* = 25 full articles met the inclusion criteria. However, of those *n* = 13 were conducted in high-income settings, *n* = 1 did not report information for children with CP separately, thus the outcome could not be differentiated from other study participants, and *n* = 3 were conducted in HICs and did not report data on CP separately. Hence, those *n* = 17 were excluded. Finally, *n* = 8 articles published between the years 2008 and 2019 were included in the review for data charting. A flow diagram of the study selection procedure is shown in Figure 1. A summary of the excluded studies is also available in Supplementary Table S2.

The details about study characteristics, study participants, intervention details, and outcomes of the included studies are summarized in the subsequent sections.

### 3.1. Study Characteristics

The characteristics of the included studies have been summarized in Table 1.

#### 3.1.1. Study Design

Five out of eight studies were experimental in design (three conducted in lower middle-income countries (lower MICs) and two in upper middle-income countries (UMICs)) [20,21,22,23,24], two followed a descriptive analytical study design [25,26], and the remaining one was a qualitative study [27].

**Table 1:** Characteristics of the included study.

Study Details	Country		Settings	Study Design	Study Period	Study Participants	Characteristics of Participating Children with CP			
	Name	Economic Classification					Number	Age (Range, Mean (SD), Median [IQR])	Sex	GMFCS Level
Donker et al., 2019 [27]	Ghana	Lower MIC	Community-based	Qualitative	July 2015–July 2016	Children with CP; Primary caregivers of children with CP	18	Range: 1 y 6 m–11 y 11 m	F: 10, M: 8	GMFCS I–II: 4; GMFCS III–V: 14
Caselli et al., 2017 [25]	Brazil	Upper MIC	Hospital/Clinic/Centre-based	Descriptive analytical	Not reported	Children with spastic quadriplegia	54	Range: 2 y 1 m–18 y 7 m	F: 20, M: 34	Not reported
Soylu et al., 2008 [23]	Turkey	Upper MIC	Hospital/Clinic/Centre-based	Experimental	May 2007–April 2008	Children with quadriplegic CP	45	Mean ± SD: 5 y 6 m ± 3 y 7 m	F: 16, M: 29	GMFCS I–II: 3; GMFCS III–V: 42
Zuurmond et al., 2018 [24]	Ghana	Lower MIC	Community-based	Experimental	February 2015–July 2016	Children with CP; Primary caregivers of children with CP	64	Range: 1 y 6 m–12 y 0 m Mean ± SD: 3 y 10 m ± 2 y 8 m	F: 26, M: 38	GMFCS I–II: 15; GMFCS III: 17; GMFCS IV–V: 32
Adams et al., 2011 [20]	Bangladesh	Lower MIC	Hospital/Clinic/Centre-based	Experimental	Not reported	Children with CP; Primary caregivers of children with CP	22	Range: 1 y 7 m–10 y 9 m; Mean ± SD:3 y 11 m ± 2 y 3 m	F: 14, M: 8	GMFCS III–V: 22
Pike T et al., 2016 [22]	South Africa	Upper MIC	Hospital/Clinic/Centre-based	Experimental	August 2007–January 2009	Children with CP; Primary caregivers of children with CP	16	Range: 1 y 0 m–18 y 0 m	F: 6, M: 10	GMFCS I–II: 1/16; GMFCS III–V: 15/16
Gracia-Contreras et al., 2014 [26]	Mexico	Upper MIC	Hospital/Clinic/Centre-based	Descriptive analytical	2010–2011 [month not reported]	Children with quadriplegic CP	13	Range: 6 y 11 m–12 y 10 m Mean: 9 y 7 m	F: 8, M: 5	GMFCS V: 13
Omar et al.,2017 [21]	Egypt	Lower MIC	Hospital/Clinic/Centre-based	Experimental	January 2017–April 2017	Children with CP; Primary caregivers of children with CP	20	Median [IQR]: 2 y 6 m [1 y 1 m, 4 y 10 m]	F: 6, M: 14	GMFCS III-1/20; GMFCS level IV: 8/20; GMFCS level V: 11/20

CP, Cerebral Palsy; MIC, Middle-income country; GMFCS, Gross Motor Function Classification System.

#### 3.1.2. Study Location

Of the eight studies, *n* = 4 were from UMICs (Brazil, South Africa, Turkey, Mexico) [22,23,25,26], and *n* = 4 were from lower MICs (Bangladesh, Ghana × 2, Egypt) [20,21,24,27]; no studies from low-income countries (LICs) were identified. As per the geographical distribution, *n* = 3 studies were conducted in sub-Saharan Africa (Ghana × 2 and South Africa) [22,24,27], *n* = 2 were from Latin America and the Caribbean region (Brazil and Mexico) [25,26], *n* = 1 from the South Asia region (Bangladesh) [20], *n* = 1 from the Middle East and the North Africa region (Egypt) [21], and *n* = 1 from the Europe and Central Asia region (Turkey) [23].

#### 3.1.3. Study Settings

Six of the eight studies were conducted in hospital/clinic/institution-based settings [20,21,22,23,25,26]. The remaining two were conducted in community-based settings (both evaluated the impact of the same intervention model on different outcome measures) in Ghana; one followed an experimental study design and the other a qualitative approach [24,27]. Among the hospital/clinic/institution-based studies, four were experimental studies conducted in Bangladesh, Egypt, South Africa, and Turkey [20,21,22,23], and two were descriptive analytical studies conducted in Brazil and Mexico [25,26].

#### 3.1.4. Study Participants and Sample Size

Five studies had children with CP and their primary caregivers as study participants [20,21,22,24,27], and three included children with quadriplegic CP only [23,25,26].

A total of 252 children with CP were included in the selected studies (age ranged between 1 year (y) and 18 y 7months (m)). The lowest sample size was *n* = 13 (a descriptive analytical study conducted in hospital/clinic/institution-based settings in Mexico [26], and the highest sample size was *n* = 64 (a community-based experimental study conducted in Ghana) [24].

Overall, 12%, *n* = 23/198 children, had GMFCS level I–II and the 88%, *n* = 175/198 children, had GMFCS level III–V [20,21,22,23,26,27]; one study did not report the GMFCS level of the participants [24,25].

A summary of individual study design, locations, settings, participants, and sample size is presented in Table 1.

### 3.2. Intervention Details

Of the eight studies, *n* = 6 had single type interventions [20,21,23,24,25,27] and the remaining *n* = 2 had multiple interventions [22,26]. Of all, *n* = 4 provided training to parents/caregivers [20,21,24,27] and *n* = 2 involved surgical interventions (e.g., gastrostomy or nasogastric tube feeding) [25,26]. Furthermore, dietary modification (e.g., modification of calorie and nutrient density/balanced diet/frequency/consistency/nutritional adequacy) was an intervention component in *n* = 5 studies [20,21,22,23,26]. Both studies with surgical interventions were descriptive in design (i.e., comparative study, before-after study, case series) and were conducted in Brazil [25] and Mexico [26]. The studies that emphasized parental/caregiver training covered a wide range of content, such as therapy, feeding skills, dietary modifications, position, and carrying [20,21,22,24,27]. The number of sessions varied between 5–11 [20,21,22,24], and the follow-up duration ranged between 1–18 months [20,21,22,23,24,26,27]. The study implementation team was documented only in one study by Pike et al. (2016) where a team of physiotherapists, occupational therapists, and speech-language therapists provided need-based neurodevelopmental therapy (NDT) feeding intervention to children with CP, trained the caregivers on recommended NDT feeding intervention and dietary modifications for children with CP in a hospital-based/institution-based setting in South Africa [22].

The details of different interventions provided in each of the studies included in this review are summarized in Table 2.

### 3.3. Outcome Measures

Different anthropometric measurements were used to evaluate changes in the nutritional status of participating children. The most commonly used anthropometric measurements in the selected articles were weight (*n* = 8) [20,21,22,23,24,25,26,27], length/height (*n* = 7) [21,22,23,24,25,26,27], mid-upper arm circumference (MUAC) (*n* = 7) [20,21,22,23,24,26,27], and at least one skin-fold thickness measurement (*n* = 4) [22,23,25,26].

In all studies, nutritional status was determined by comparing the anthropometric measurements of children with CP to national standards/general population/WHO reference population [20,21,22,23,24,25,26,27]. However, one study also compared the anthropometric measurements with CP specific growth charts [23].

All but one article had other outcome measures in addition to the nutritional assessment; these include (i) dietary intake practices (*n* = 3) [20,25,27], feeding skills/feeding practices/feeding profiles (*n* = 5) [20,21,22,26,27], outcomes related to caregivers’ quality of life, knowledge and confidence about child care, perception about child’s health, feelings about child’s feeding difficulties, and compliance with the training [20,24], and child’s health-related quality of life [22]. Any adverse outcomes related to intervention were reported in *n* = 3 articles [20,23,24]; these outcomes included infections (one study) [23], chest health (one study) [20], and mortality (two studies) [20,24] (Table 3).

### 3.4. Effect of Different Interventions on Nutritional Status of Children with CP

Of the eight studies, seven included follow-up data of the participating children with CP (i.e., two or more data points for each participant) [20,21,22,23,24,26,27] and the other one compared between two groups (i.e., intervention vs. control group) [25]. Of those *n* = 7 studies with longitudinal data [20,21,22,23,24,26,27], *n* = 4 showed improvement in nutritional status among children with CP following intervention [20,22,23,26], one showed no change [21], whereas two showed deterioration [24,27] in the nutritional status following respective interventions among children with CP in selected LMICs.

Four out of the five studies that focused on dietary modifications showed improvement in nutritional status of participating children [20,22,23,26]. Studies that focused on improving feeding skills (*n* = 3) had both positive and negative nutritional outcomes [20,21,24].

Both studies with GTF as an intervention showed a positive effect of GTF on the nutritional outcome of children with CP [25,26], however, of those none reported any adverse outcome of GTF [25,26]. (Table 3)

## 4. Discussion

In this review we provide a summary of the available evidence on different nutrition interventions for children with CP in LMICs. Our search identified only a few relevant studies on the topic that were conducted in resource-constrained settings of LMICs. Only eight articles were identified, none of which had representation from low-income countries. Such an evidence gap from LMICs where the majority of children with CP reside is concerning [11]. Considering the high burden of malnourished children with CP in LMICs [1,2,3,4,5,8,9], there is a dire need to reduce this evidence gap, and integration of nutrition interventions in the existing service plan for children with CP is essential.

There are global efforts toward reducing the burden of malnutrition among children, especially in LMICs. The United Nations Sustainable Development Goals (UN SDGs) has an emphasis on childhood nutrition for human growth and development [28]. The SDGs also strongly advocate for disability inclusiveness, especially the SDGs which are related to growth, health, education, employment, and addressing inequality globally [29]. National and international partners are working together to develop standard guidelines, strategies, and interventions addressing the immediate determinants (i.e., nutrition-specific intervention) and the underlying or root causes (i.e., nutrition-sensitive interventions) to improve the overall nutritional status of children, especially in LMICs [30,31]. These interventions should be disability-inclusive, and priority should be given to reporting/documentation of the effectiveness of different nutrition interventions on growth and nutrition of children with disabilities, including CP.

While reviewing the limited available studies, we observed that a majority of the interventions were trialed or implemented in institution-based settings. Though interventions implemented in the institution-based settings have been proven to result in better outcomes in the past [6], our evidence suggests the majority of children with CP in LMICs lack access to any rehabilitation services for numerous reasons, including limited availability of services in their neighborhoods, transport difficulties, and financial constraints [12,32]. Furthermore, the severe shortage of trained health professionals, e.g., rehabilitation service providers and dietitians, limits the scope to implement and scale-up different institution-based interventions for children with CP in LMICs [13]. In such a context, community-based approaches are highly recommended and there is a lack of evidence on community-based nutrition interventions for children with CP in LMICs. Nevertheless, in one recent study from Bangladesh, community-based parent-led intervention was found to be highly successful in improving functional outcomes of children with CP [33]. Emphasis should be given to the capacity development of mid-level service providers on growth monitoring and promotion, early identification, different blanket interventions, and referral to prevent and treat malnutrition among children with CP in LMICs.

In addition to the settings, the sample sizes of the studies were relatively low. Although the included studies provided valuable insights on different nutritional interventions, the small sample size limits the strength and generalizability of the study findings. Moreover, most children with CP in the selected studies had severe functional motor limitations. These could be due to selection bias related to more severe institutional samples, as well as the more severe end of the clinical spectrum of CP in LMICs, likely exacerbated by delayed age of diagnosis and lack of access to rehabilitation services [12,32,34]. Studies also show that the likelihood of receiving intervention and rehabilitation is higher among those with severe CP (e.g., GMFCS level III–V) compared to milder forms (e.g., GMFCS I–II) [12]. However, it is also known that severe functional motor limitations (e.g., GMFCS level III–V) in children with CP are often accompanied by severe oromotor dysfunction, e.g., dysphagia [35]. It remains unclear whether the interventions for children with more severe motor limitations are also appropriate for children with CP who are GMFCS I–II.

Most of the interventions identified in this review improved the nutritional status of participating children with CP. Importantly, nutrition-specific interventions (i.e., interventions that aim to intervene in or correct the immediate causes of malnutrition, such as inadequate diet, disease severity and caring practices) had comparatively better outcomes than other intervention strategies [20,22,24,30]. We observed that the interventions that included dietary modifications (only, or as a component of multiple approaches) had better effect on nutritional outcomes of children with CP. Studies where participants received surgical interventions (e.g., GTF) also showed a positive effect, whereas studies that provided behavioral intervention on feeding skills only did not show any significant change.

To the best of our knowledge, this is one of the first reviews conducted on nutrition interventions for children with CP in LMICs. Although during our search we identified several studies that aimed to improve the nutritional status of children with CP in HICs, our search revealed only a few reviews (including one systematic review) that summarized findings from different nutrition intervention studies among children with CP in HICs [6,36,37]. The data provided in our study, therefore, contribute important information to the existing knowledge gap. Nevertheless, this review has several limitations. We focused our search on two databases, so a risk remains that there may have been other publications available through other databases. However, our search revealed a large number of duplicates; therefore, we are confident that we have covered all or almost all of the studies published online. Second, we only searched for articles that had our key words in title/abstracts/author specified key words in the articles. This may have reduced the number of articles initially identified for title screening. Third, we did not assess the risk of bias and quality of evidence of the articles finally included in the review, which may have posed bias in the information reported. Finally, due to the differences in reporting format and data availability, we could only include a few common variables and could not report on some of the important characteristics of the participants. For the same reason, we had to rely on reporting the descriptive findings only.

## 5. Conclusions

The current review highlights the knowledge gap and lack of available evidence on nutrition interventions for children with CP in LMICs. Strong evidence is essential to identify and determine the best practices and nutritional management guidelines for children with CP, especially for optimal utilization of the limited available resources in LMICs. National and international stakeholders, therefore, should make this a priority for future research and services. Considering the high proportion of children with CP with undernutrition in LMICs, existing interventions and services for children with CP should integrate a nutrition component. The current limited evidence suggests that nutrition-specific programs have more of a positive effect on the growth of children with CP than other strategies. This should be taken under consideration when planning for any nutrition focus and other comprehensive services for children with CP in LMICs.

## Figures and Tables

**Table 2:** Details of different interventions provided in the included studies.

Study Details	Intervention Provided				
	Type	Settings	Contents	Number of Sessions	F/U Period
Donker et al., 2019 [27]	Training to parents/caregivers	Community	Group support sessions followed the “Getting to Know Cerebral Palsy” (GTKCP) manual comprised of 11 modules including a module on “Eating and Drinking”	Not reported	12 months
Caselli et al., 2017 [25]	Gastrostomy tube placement/feeding/nasogastric tube feeding	Hospital/clinic/centre	A comparison between children fed by gastrostomy (GTF) at least for 6 months versus children fed orally (PO)	Not applicable	Not applicable
Soylu et al., 2008 [23]	Nutritional rehabilitation/therapy	Hospital/clinic/centre	Dietary modification	Not reported	6 months
Zuurmond et al., 2018 [24]	Training to parents/caregivers	Community	Evaluation of the child, positioning and carrying, communication, everyday activities, feeding child, play, disability in the local community, running an own parent support group, assistive devices and resources	Monthly 3 h sessions, total 11 sessions for each participant	11 months
Adams et al., 2012 [20]	Training to parents/caregivers	Hospital/clinic/centre	(i) Dietary modification (calorie density, balanced, frequency, portion size), (ii) Feeding skills (posture/positioning, responsive, self-feeding)	6 fortnightly training sessions each pair	4–6 months
Pike T et al., 2016 [22]	Neurodevelopmental therapy (NDT) feeding intervention	Hospital/clinic/centre	NDT: speech-language therapy, physiotherapy, occupational therapy, assistive device	90 min sessions for 5 consecutive days	18 months
	Training to parents/caregivers		Caregiver training on the recommended therapies as part of NDT		
	Dietary modification				
Gracia-Contreras et al., 2014 [26]	Gastrostomy tube placement/feeding/nasogastric tube feeding	Hospital/clinic/centre	Not applicable	Not applicable	4 weeks
	Dietary modification		Calorie and nutrient (e.g., protein adjustments)		
Omar et al., 2017 [21]	Training to parents/caregivers	Hospital/clinic/centre	(i) Feeding skills (position, mode of feeding, utensils, associated feeding problems), (ii) responsive feeding, (iii) food consistency and adequacy.	10 sessions over 5 days, 3 h each day	3 months

**Table 3:** The outcome of different interventions provided in the included studies.

Study Details	Nutritional Outcome Measures		Other Outcome Measures	Adverse Outcome Reported	Change in Nutritional Indicators		Nutritional Status Improved
	Anthropometric Measurements	Indicator			Pre-Intervention/Intervention Group	Post Intervention/Control Group	
Donker et al., 2019 [27]	Weight	N/A	(i) Changes in positioning, utensils, and feeding skills, (ii) Dietary intake	Not reported	N/A	N/A	No ^1^
	Length/Height	HAZ			Mean: −2.13	Mean: −2.62	
		Stunting			50%	50%	
	Weight/Height	WHZ			Mean: −2.47	Mean: −2.24	
		Wasting			50%	50%	
	MUAC	Mean			Mean: 151 mm	Mean: 149 mm	
Caselli et al., 2017 [25]	Weight	WA (percentiles)	(i) Dietary intake	Not reported	GTF: <25th 20.0%; 25–90th 68.0%; >90th 12.0%	PO: <25th 24.1%; 25–90th 72.4%, 90th 3.4%	N/A ^2^
	Length/Height, KH	HA (percentiles)			GTF: 10–90th 10.0%	PO: 10–90th 100.0%	
	Weight/Height	BA (percentiles)			GTF: <5th 20.0%; 10–90th 72.0%; >90th 8.0%	PO: <5th 24.1%; 10–90th 72.4%; >90th 3.4%	
	AC	Percentile			GTF: <10th 40%; 10–90th and >95th 60%	PO: <10th 75.8%; 10–90th and >95th 24.1%	
	TSF	Percentile			GTF: <10th 12%; 10–90th 64%; >95th 24%	PO: <10th 62.7%; 10–90th 37.9%; >95th 0%	
	AMC	Percentile			GTF: <10th 52%; 10–90th and >95th 48%	PO: <10th 68.9%; 10–90th and >95th 31%	
	AMA	Percentile			GTF: <10th 60%; 10–90th and >95th 40%	PO: <10th 72.4%; 10–90th and >95th 27.5%	
	AAA	Percentile			GTF: <5th 16%; 10–90th and >95th 84%	PO: <5th 55.1%; 10–90th and >95th 83%	
Soylu et al., 2008 [23]	Weight	WAZ	None	Number of infections	Mean ± SD: −2.1 ± 0.9	Mean ± SD: −1.8 ± 0.9	Yes ^3^
	Length/Height	HAZ			Mean ± SD: −2.4 ± 2.1	Mean ± SD: −2.3 ± 2.0	
	Weight/Height	BMI			Mean ± SD: 13.6 ± 2.1 kg/m^2^	Mean ± SD: 14.4 ± 2.0 kg/m^2^	
		WH (NCHS percentiles)			<10th 18/31; 10–50th 9/31;50–90th 0/31; >90th 4/31	<10th 14/31; 10–50th 9/31;50–90th 5/31; >90th 3/31	
	MUAC	Mean, SD			Mean ± SD: 14.5 ± 2.2 cm	Mean ± SD: 15.2 ± 2.2 cm	
	TSF	Mean, SD			Mean ± SD: 9.8 ± 3.5 mm	Mean ± SD: 10.4 ± 4.1 mm	
	Not applicable	Not applicable			They also compared the findings with CP growth chart. Significant improvement after therapies was observed for weight, height, MUAC, TSF, WAZ, WHZ, BMI		
Zuurmond et al., 2018 [24]	Weight	WAZ; Underweight	(i) Caregiver’s quality of life, (ii) Caregiver’s knowledge, confidence about providing care to their child, (iii) Caregiver’s perception about child’s physical and emotional health	(i) Serious health problem/illness in preceding 6 months, (ii) Mortality	Mean (95% CI): −2.6 (−2.9, −2.2);Underweight: 24.5%, Severely underweight: 38.5%	Mean (95% CI): −2.8 (−3.1, −2.3);Underweight: 17.2%, Severely underweight: 48.2%	No ^4^
	Length/Height	HAZ; Stunting			Mean (95% CI): −2.3 (−2.6, −1.9);Stunted: 26.9%, Severely stunted: 26.9%	Mean (95% CI): −2.7 (−3.0, −2.4);Stunted: 25.0%, Severely stunted: 39.1%	
	Weight/height	WHZ; wasting			Mean (95% CI): −2.1 (−2.5, −1.6);Wasted: 40.0%, severely wasted: 20.0%	Mean (95% CI): −1.9 (−2.5, −1.3);Wasted: 12.9%, severely wasted: 32.3%	
	MUAC	Mean, 95% CI; Wasting			144.8 (139.3, 150.4)Wasted: 15.2%, Severely wasted: 0%	144.4 (137.9, 150.8); Wasted: 13.0%, Severely wasted: 8.7%	
Adams et al., 2011 [20]	Weight	WAZ	(i) Dietary intake, (ii) Child’s feeding skills and mood, (iii) Caregivers’ feelings about child’s feeding difficulties, (iv) Caregiver’s compliance with training recommendations, (v) Child and caregiver’s behavior during mealtime	(i) Chest health, (ii) Mortality	Mean ± SD: −4.83 ± 1.84	Mean ± SD: −4.07 ± 2.45	Yes ^5^
	MUAC	Mean, SD			Mean ± SD: 14.75 ± 1.41 cm	Mean ± SD: 15.46 ± 1.57)	
Pike T et al., 2016 [22]	Weight	Mean, SD, median	(i) Child’s health related quality of life, (ii) Feeding profile	Not reported	Mean: 16.0 (4.0) kg; Median: 15.5 kg	Mean: 18.3 (5.1) kg; Median: 17.8 kg	Yes ^6^
	Length/Height				Mean: 101.9 (15.2) cm; Median: 100.6 cm	Mean: 106.7 (13.5) cm; Median: 105.1 cm	
	MUAC				Mean: 17.5 (1.6) cm; Median: 17.8 cm	Mean: 18.3 (2.1) cm; Median: 18.1 cm	
	TSF				Mean: 7.7 (3.0) mm; Median: 7.3 mm	Mean: 8.6 (3.7) mm; Median: 7.8 mm	
Gracia-Contreras et al., 2014 [26]	Weight	Mean, SD	(i) Bioelectrical Impedance analysis-fat mass and fat free mass, (ii) Feeding type and energy intake	Not reported	Mean ± SD: 11.9 ± 2.3 kg	Mean ± SD: 14.6 ± 2.6 kg	Yes ^7^
	Weight/ Height	BMI			Mean ± SD: 10.0 ± 1.0 kg/m^2^	Mean ± SD: 12 ± 0.9 kg/m^2^	
		BAZ			Mean ± SD: −2.8 ± 0.5	Mean ± SD: −1.9 ± 0.3	
	MUAC	MUACZ			Mean ± SD: −3.5 ± 0.3	Mean ± SD: −2.8 ± 0.4	
	TSF	Mean, SD			Mean ± SD: 4.0 ± 1.8 mm	Mean ± SD: 7.4 ± 2.7 mm	
		TSFZ			Mean ± SD: 7.4 ± 2.7	Mean ± SD: −0.9 ± 0.4	
	SSF	Mean, SD			Mean ± SD: 3.7 ± 0.5 mm	Mean ± SD: 6.4 ± 1.9 mm	
		SSFZ			Mean ± SD: −0.8 ± 0.1	Mean ± SD: −0.3 ± 0.5	
	THSF	Mean, SD			Mean ± SD: 7.3 ± 5.3 mm	Mean ± SD: 13.5 ± 7.7 mm	
	CSF	Mean, SD			Mean ± SD: 5.8 ± 3.4 mm	Mean ± SD: 9.4 ± 4.9 mm	
	AMA	Mean, SD			Mean ± SD: 8.0 ± 1.2 cm^2^	Mean ± SD: 9.4 ± 1.4 cm^2^	
	AFA	Mean, SD			Mean ± SD: 2.2 ± 1.1 cm^2^	Mean ± SD: 4.5 ± 1.8 cm^2^	
Omar et al., 2017 [21]	Weight	Mean, SD	(i) Feeding problem, (ii) feeding practices	Not reported	Mean ± SD: 10.1 ± 2.4 kg	Mean ± SD: 10 ± 2.5 kg	No change ^8^
		WAZ			Mean ± SD: −3.36 ± 1.26	Mean ± SD: −3.36 ± 1.26	
	Length/Height	Mean, SD			Mean ± SD: 84.80 ± 10.26 cm	Mean ± SD: 84.80 ± 10.26 cm	
		BMI			Mean ± SD: 14.77 ± 2.08 kg/m^2^	Mean ± SD: 14.77 ± 2.08 kg/m^2^	
	MUAC	Mean, SD			Mean ± SD: 14.75 ± 1.81 cm	Mean ± SD: 14.75 ± 1.81 cm	
	TL	Mean, SD			Mean ± SD: 16.8 ± 3.03 cm	Mean ± SD: 16.8 ± 3.03 cm	

AAA: Adipose Arm Area; AC: Arm circumference; AFA: Arm Fat Area; AMA: Arm Muscle Area; AMC: Arm Muscle Circumference; BA: BMI for age; BAZ: BMI for age z score; BMI: Body mass index; CSF: Calf skin fold; CSFZ: Calf skin fold z score; HA: height for age; HAZ: height for age z score; KH: knee height; MUAC: mid-upper arm circumference; MUACZ: MUAC for age z score; SSF: subscapular skinfold thickness; SSFZ: SSF for age z score; THSF: Thigh skin fold; THSFZ: THSF for age z score; TSF: triceps skinfold thickness; TSFZ: TSF for age z score; WA: weight for age; WAZ: weight for age z score; WH: weight for height; WHZ: weight for height z score. N/A: Not applicable. ^1^ *p* value was not reported; ^2^ *p* values for the differences between two groups were *p* = 0.05 for WA, *p* = 0.09 for BA, *p* = 0.01 for AC, *p* < 0.001 for TSF, *p* = 0.20 for AMC, *p* = 0.33 for AMA and *p* = 0.003 for AAA; ^3^ *p* < 0.001 for changes in WAZ, *p* = 0.962 for changes in HAZ, *p* < 0.001 for changes in MUAC, *p* = 0.004 for changes in TSF, *p* = 0.008 for changes in BMI; ^4^ *p* = 0.08 for changes in WAZ, *p* = 0.003 for changes in HAZ, *p* = 0.24 for changes in WHZ, *p* = 0.8; ^5^ *p* = 0.02 for changes in WAZ, *p* = 0.001 for changes in MUAC; ^6^ *p* value was not reported pre-intervention and last follow-up; ^7^ *p* < 0.001 for changes in BMI, BAZ, MUACZ, TSF, TSFZ, SSF, AMA, AFA; *p* < 0.01 for changes in THSFZ and SSFZ; ^8^ *p* = 1.00 for all indicators.

## Data Availability

All data have been presented in the manuscript and no additional data are available to access.

## Supplementary Materials

The following are available online at https://www.mdpi.com/article/10.3390/nu14061211/s1, Table S1: Database search history (Scopus); Table S2: Summary of the study characteristics of the studies excluded after full article review and reason for exclusion [38,39,40,41,42,43,44,45,46,47,48,49,50,51,52,53,54].
