# Supplementary material for: Nutrition Interventions for Children with Cerebral Palsy in Low- and Middle-Income Countries: A Scoping Review
Source: Nutrients. 2022 Mar 12;14(6):1211. doi: 10.3390/nu14061211 (PMC8951851; doi:10.3390/nu14061211)
Supplement: Supplementary file 1 [file nutrients-14-01211-s001.zip › nutrients-1610968-supplementary.pdf]

Table S1: Database search history

Database: Scopus
Search strategy

SL	Search details	No of articles
1.	(TITLE-ABS-KEY ("cerebral palsy") AND TITLE-ABS-KEY (child*) AND TITLE-ABS-KEY (educat*) AND TITLE-ABS-KEY (nutri*))	(77)
2.	(TITLE-ABS-KEY ("cerebral palsy") AND TITLE-ABS-KEY (child*) AND TITLE-ABS-KEY (educat*) AND TITLE-ABS-KEY (malnutri*))	(28)
3.	(TITLE-ABS-KEY ("cerebral palsy") AND TITLE-ABS-KEY (child*) AND TITLE-ABS-KEY (educat*) AND TITLE-ABS-KEY (growth))	(122)
4.	(TITLE-ABS-KEY ("cerebral palsy") AND TITLE-ABS-KEY (child*) AND TITLE-ABS-KEY (educat*) AND TITLE-ABS-KEY (health))	(965)
5.	(TITLE-ABS-KEY ("cerebral palsy") AND TITLE-ABS-KEY (child*) AND TITLE-ABS-KEY (feed*) AND TITLE-ABS-KEY (nutri*))	(438)
6.	(TITLE-ABS-KEY (cerebral AND palsy) AND TITLE-ABS-KEY (child*) AND TITLE-ABS-KEY (feed*) AND TITLE-ABS-KEY (malnutri*))	(155)
7.	(TITLE-ABS-KEY (cerebral AND palsy) AND TITLE-ABS-KEY (child*) AND TITLE-ABS-KEY (feed*) AND TITLE-ABS-KEY (growth))	(241)
8.	(TITLE-ABS-KEY (cerebral AND palsy) AND TITLE-ABS-KEY (child*) AND TITLE-ABS-KEY (feed*) AND TITLE-ABS-KEY (health))	(344)
9.	(TITLE-ABS-KEY (cerebral AND palsy) AND TITLE-ABS-KEY (child*) AND TITLE-ABS-KEY (surg*) AND TITLE-ABS-KEY (nutri*))	(142)
10.	(TITLE-ABS-KEY (cerebral AND palsy) AND TITLE-ABS-KEY (child*) AND TITLE-ABS-KEY (surg*) AND TITLE-ABS-KEY (malnutri*))	(44)
11.	(TITLE-ABS-KEY (cerebral AND palsy) AND TITLE-ABS-KEY (child*) AND TITLE-ABS-KEY (surg*) AND TITLE-ABS-KEY (growth))	(293)
12.	(TITLE-ABS-KEY (cerebral AND palsy) AND TITLE-ABS-KEY (child*) AND TITLE-ABS-KEY (surg*) AND TITLE-ABS-KEY (health))	(571)
13.	(TITLE-ABS-KEY (cerebral AND palsy) AND TITLE-ABS-KEY (child*) AND TITLE-ABS-KEY (techn*) AND TITLE-ABS-KEY (nutri*))	(104)
14.	(TITLE-ABS-KEY (cerebral AND palsy) AND TITLE-ABS-KEY (child*) AND TITLE-ABS-KEY (techn*) AND TITLE-ABS-KEY (malnutri*))	(25)
15.	(TITLE-ABS-KEY (cerebral AND palsy) AND TITLE-ABS-KEY (child*) AND TITLE-ABS-KEY (techn*) AND TITLE-ABS-KEY (growth))	(181)
16.	(TITLE-ABS-KEY (cerebral AND palsy) AND TITLE-ABS-KEY (child*) AND TITLE-ABS-KEY (techn*) AND TITLE-ABS-KEY (health))	(520)
17.	(TITLE-ABS-KEY (cerebral AND palsy) AND TITLE-ABS-KEY (child*) AND TITLE-ABS-KEY (train*) AND TITLE-ABS-KEY (nutri*))	(51)
18.	(TITLE-ABS-KEY (cerebral AND palsy) AND TITLE-ABS-KEY (child*) AND TITLE-ABS-KEY (train*) AND TITLE-ABS-KEY (malnutri*))	(19)
19.	(TITLE-ABS-KEY (cerebral AND palsy) AND TITLE-ABS-KEY (child*) AND TITLE-ABS-KEY (train*) AND TITLE-ABS-KEY (growth))	(66)
20.	(TITLE-ABS-KEY (cerebral AND palsy) AND TITLE-ABS-KEY (child*) AND TITLE-ABS-KEY (train*) AND TITLE-ABS-KEY (health))	(526)

SL	Search details	No of articles
21.	(TITLE-ABS-KEY (cerebral AND palsy) AND TITLE-ABS-KEY (adolescent*) AND TITLE-ABS-KEY (educat*) AND TITLE-ABS-KEY (nutri*))	(30)
22.	(TITLE-ABS-KEY (cerebral AND palsy) AND TITLE-ABS-KEY (adolescent*) AND TITLE-ABS-KEY (educat*) AND TITLE-ABS-KEY (malnutri*))	(8)
23.	(TITLE-ABS-KEY (cerebral AND palsy) AND TITLE-ABS-KEY (adolescent*) AND TITLE-ABS-KEY (educat*) AND TITLE-ABS-KEY (growth))	(25)
24.	(TITLE-ABS-KEY (cerebral AND palsy) AND TITLE-ABS-KEY (adolescent*) AND TITLE-ABS-KEY (educat*) AND TITLE-ABS-KEY (health))	(381)
25.	(TITLE-ABS-KEY (cerebral AND palsy) AND TITLE-ABS-KEY (adolescent*) AND TITLE-ABS-KEY (feed*) AND TITLE-ABS-KEY (nutri*))	(170)
26.	(TITLE-ABS-KEY (cerebral AND palsy) AND TITLE-ABS-KEY (adolescent*) AND TITLE-ABS-KEY (feed*) AND TITLE-ABS-KEY (malnutri*))	(58)
27.	(TITLE-ABS-KEY (cerebral AND palsy) AND TITLE-ABS-KEY (adolescent*) AND TITLE-ABS-KEY (feed*) AND TITLE-ABS-KEY (growth))	(80)
28.	(TITLE-ABS-KEY (cerebral AND palsy) AND TITLE-ABS-KEY (adolescent*) AND TITLE-ABS-KEY (feed*) AND TITLE-ABS-KEY (health))	(113)
29.	(TITLE-ABS-KEY (cerebral AND palsy) AND TITLE-ABS-KEY (adolescent*) AND TITLE-ABS-KEY (surg*) AND TITLE-ABS-KEY (nutri*))	(69)
30.	(TITLE-ABS-KEY (cerebral AND palsy) AND TITLE-ABS-KEY (adolescent*) AND TITLE-ABS-KEY (surg*) AND TITLE-ABS-KEY (malnutri*))	(17)
31.	(TITLE-ABS-KEY (cerebral AND palsy) AND TITLE-ABS-KEY (adolescent*) AND TITLE-ABS-KEY (surg*) AND TITLE-ABS-KEY (growth))	(93)
32.	(TITLE-ABS-KEY (cerebral AND palsy) AND TITLE-ABS-KEY (adolescent*) AND TITLE-ABS-KEY (surg*) AND TITLE-ABS-KEY (health))	(283)
33.	(TITLE-ABS-KEY (cerebral AND palsy) AND TITLE-ABS-KEY (adolescent*) AND TITLE-ABS-KEY (techn*) AND TITLE-ABS-KEY (nutri*))	(44)
34.	(TITLE-ABS-KEY (cerebral AND palsy) AND TITLE-ABS-KEY (adolescent*) AND TITLE-ABS-KEY (techn*) AND TITLE-ABS-KEY (malnutri*))	(10)
35.	(TITLE-ABS-KEY (cerebral AND palsy) AND TITLE-ABS-KEY (adolescent*) AND TITLE-ABS-KEY (techn*) AND TITLE-ABS-KEY (growth))	(45)
36.	(TITLE-ABS-KEY (cerebral AND palsy) AND TITLE-ABS-KEY (adolescent*) AND TITLE-ABS-KEY (techn*) AND TITLE-ABS-KEY (health))	(200)
37.	(TITLE-ABS-KEY (cerebral AND palsy) AND TITLE-ABS-KEY (adolescent*) AND TITLE-ABS-KEY (train*) AND TITLE-ABS-KEY (nutri*))	(17)
38.	(TITLE-ABS-KEY (cerebral AND palsy) AND TITLE-ABS-KEY (adolescent*) AND TITLE-ABS-KEY (train*) AND TITLE-ABS-KEY (malnutri*))	(4)
39.	(TITLE-ABS-KEY (cerebral AND palsy) AND TITLE-ABS-KEY (adolescent*) AND TITLE-ABS-KEY (train*) AND TITLE-ABS-KEY (growth))	(13)
40.	(TITLE-ABS-KEY (cerebral AND palsy) AND TITLE-ABS-KEY (adolescent*) AND TITLE-ABS-KEY (train*) AND TITLE-ABS-KEY (health))	(225)
41.	(TITLE-ABS-KEY (cerebral AND palsy) AND TITLE-ABS-KEY (infant) AND TITLE-ABS-KEY (educat*) AND TITLE-ABS-KEY (nutri*))	(29)

SL	Search details	No of articles
42.	(TITLE-ABS-KEY (cerebral AND palsy) AND TITLE-ABS-KEY (infant) AND TITLE-ABS-KEY (educat*) AND TITLE-ABS-KEY (malnutri*))	(13)
43.	(TITLE-ABS-KEY (cerebral AND palsy) AND TITLE-ABS-KEY (infant) AND TITLE-ABS-KEY (educat*) AND TITLE-ABS-KEY (growth))	(89)
44.	(TITLE-ABS-KEY (cerebral AND palsy) AND TITLE-ABS-KEY (infant) AND TITLE-ABS-KEY (educat*) AND TITLE-ABS-KEY (health))	(313)
45.	(TITLE-ABS-KEY (cerebral AND palsy) AND TITLE-ABS-KEY (infant) AND TITLE-ABS-KEY (feed*) AND TITLE-ABS-KEY (nutri*))	(197)
46.	(TITLE-ABS-KEY (cerebral AND palsy) AND TITLE-ABS-KEY (infant) AND TITLE-ABS-KEY (feed*) AND TITLE-ABS-KEY (malnutri*))	(40)
47.	(TITLE-ABS-KEY (cerebral AND palsy) AND TITLE-ABS-KEY (infant) AND TITLE-ABS-KEY (feed*) AND TITLE-ABS-KEY (growth))	(150)
48.	(TITLE-ABS-KEY (cerebral AND palsy) AND TITLE-ABS-KEY (infant) AND TITLE-ABS-KEY (feed*) AND TITLE-ABS-KEY (health))	(147)
49.	(TITLE-ABS-KEY (cerebral AND palsy) AND TITLE-ABS-KEY (infant) AND TITLE-ABS-KEY (surg*) AND TITLE-ABS-KEY (nutri*))	(64)
50.	(TITLE-ABS-KEY (cerebral AND palsy) AND TITLE-ABS-KEY (infant) AND TITLE-ABS-KEY (surg*) AND TITLE-ABS-KEY (malnutri*))	(11)
51.	(TITLE-ABS-KEY (cerebral AND palsy) AND TITLE-ABS-KEY (infant) AND TITLE-ABS-KEY (surg*) AND TITLE-ABS-KEY (growth))	(90)
52.	(TITLE-ABS-KEY (cerebral AND palsy) AND TITLE-ABS-KEY (infant) AND TITLE-ABS-KEY (surg*) AND TITLE-ABS-KEY (health))	(130)
53.	(TITLE-ABS-KEY (cerebral AND palsy) AND TITLE-ABS-KEY (infant) AND TITLE-ABS-KEY (techn*) AND TITLE-ABS-KEY (nutri*))	(45)
54.	(TITLE-ABS-KEY (cerebral AND palsy) AND TITLE-ABS-KEY (infant) AND TITLE-ABS-KEY (techn*) AND TITLE-ABS-KEY (malnutri*))	(7)
55.	(TITLE-ABS-KEY (cerebral AND palsy) AND TITLE-ABS-KEY (infant) AND TITLE-ABS-KEY (techn*) AND TITLE-ABS-KEY (growth))	(88)
56.	(TITLE-ABS-KEY (cerebral AND palsy) AND TITLE-ABS-KEY (infant) AND TITLE-ABS-KEY (techn*) AND TITLE-ABS-KEY (health))	(172)
57.	(TITLE-ABS-KEY (cerebral AND palsy) AND TITLE-ABS-KEY (infant) AND TITLE-ABS-KEY (train*) AND TITLE-ABS-KEY (nutri*))	(10)
58.	(TITLE-ABS-KEY (cerebral AND palsy) AND TITLE-ABS-KEY (infant) AND TITLE-ABS-KEY (train*) AND TITLE-ABS-KEY (malnutri*))	(6)
59.	(TITLE-ABS-KEY (cerebral AND palsy) AND TITLE-ABS-KEY (infant) AND TITLE-ABS-KEY (train*) AND TITLE-ABS-KEY (growth))	(28)
60.	(TITLE-ABS-KEY (cerebral AND palsy) AND TITLE-ABS-KEY (infant) AND TITLE-ABS-KEY (train*) AND TITLE-ABS-KEY (health))	(115)
61.	(TITLE-ABS-KEY (cerebral AND palsy) AND TITLE-ABS-KEY (toddler) AND TITLE-ABS-KEY (educat*) AND TITLE-ABS-KEY (nutri*))	(1)
62.	(TITLE-ABS-KEY (cerebral AND palsy) AND TITLE-ABS-KEY (toddler) AND TITLE-ABS-KEY (educat*) AND TITLE-ABS-KEY (malnutri*))	(0)
63.	(TITLE-ABS-KEY (cerebral AND palsy) AND TITLE-ABS-KEY (toddler) AND TITLE-ABS-KEY (educat*) AND TITLE-ABS-KEY (growth))	(8)

SL	Search details	No of articles
64.	(TITLE-ABS-KEY (cerebral AND palsy) AND TITLE-ABS-KEY (toddler) AND TITLE-ABS-KEY (educat*) AND TITLE-ABS-KEY (health))	(7)
65.	(TITLE-ABS-KEY (cerebral AND palsy) AND TITLE-ABS-KEY (toddler) AND TITLE-ABS-KEY (feed*) AND TITLE-ABS-KEY (nutri*))	(5)
66.	(TITLE-ABS-KEY (cerebral AND palsy) AND TITLE-ABS-KEY (toddler) AND TITLE-ABS-KEY (feed*) AND TITLE-ABS-KEY (malnutri*))	(1)
67.	(TITLE-ABS-KEY (cerebral AND palsy) AND TITLE-ABS-KEY (toddler) AND TITLE-ABS-KEY (feed*) AND TITLE-ABS-KEY (growth))	(3)
68.	(TITLE-ABS-KEY (cerebral AND palsy) AND TITLE-ABS-KEY (toddler) AND TITLE-ABS-KEY (feed*) AND TITLE-ABS-KEY (health))	(5)
69.	(TITLE-ABS-KEY (cerebral AND palsy) AND TITLE-ABS-KEY (toddler) AND TITLE-ABS-KEY (surg*) AND TITLE-ABS-KEY (nutri*))	(2)
70.	(TITLE-ABS-KEY (cerebral AND palsy) AND TITLE-ABS-KEY (toddler) AND TITLE-ABS-KEY (surg*) AND TITLE-ABS-KEY (malnutri*))	(0)
71.	(TITLE-ABS-KEY (cerebral AND palsy) AND TITLE-ABS-KEY (toddler) AND TITLE-ABS-KEY (surg*) AND TITLE-ABS-KEY (growth))	(3)
72.	(TITLE-ABS-KEY (cerebral AND palsy) AND TITLE-ABS-KEY (toddler) AND TITLE-ABS-KEY (surg*) AND TITLE-ABS-KEY (health))	(3)
73.	(TITLE-ABS-KEY (cerebral AND palsy) AND TITLE-ABS-KEY (toddler) AND TITLE-ABS-KEY (techn*) AND TITLE-ABS-KEY (nutri*))	(0)
74.	(TITLE-ABS-KEY (cerebral AND palsy) AND TITLE-ABS-KEY (toddler) AND TITLE-ABS-KEY (techn*) AND TITLE-ABS-KEY (malnutri*))	(0)
75.	(TITLE-ABS-KEY (cerebral AND palsy) AND TITLE-ABS-KEY (toddler) AND TITLE-ABS-KEY (techn*) AND TITLE-ABS-KEY (growth))	(1)
76.	(TITLE-ABS-KEY (cerebral AND palsy) AND TITLE-ABS-KEY (toddler) AND TITLE-ABS-KEY (techn*) AND TITLE-ABS-KEY (health))	(8)
77.	(TITLE-ABS-KEY (cerebral AND palsy) AND TITLE-ABS-KEY (toddler) AND TITLE-ABS-KEY (train*) AND TITLE-ABS-KEY (nutri*))	(1)
78.	(TITLE-ABS-KEY (cerebral AND palsy) AND TITLE-ABS-KEY (toddler) AND TITLE-ABS-KEY (train*) AND TITLE-ABS-KEY (malnutri*))	(0)
79.	(TITLE-ABS-KEY (cerebral AND palsy) AND TITLE-ABS-KEY (toddler) AND TITLE-ABS-KEY (train*) AND TITLE-ABS-KEY (growth))	(3)
80.	(TITLE-ABS-KEY (cerebral AND palsy) AND TITLE-ABS-KEY (toddler) AND TITLE-ABS-KEY (train*) AND TITLE-ABS-KEY (health))	(9)
81.	(TITLE-ABS-KEY ("neurodevelopmental disorder") AND TITLE-ABS-KEY (child*) AND TITLE-ABS-KEY (educat*) AND TITLE-ABS-KEY (nutri*))	(37)
82.	(TITLE-ABS-KEY ("neurodevelopmental disorder") AND TITLE-ABS-KEY (child*) AND TITLE-ABS-KEY (educat*) AND TITLE-ABS-KEY (malnutri*))	(7)
83.	(TITLE-ABS-KEY ("neurodevelopmental disorder") AND TITLE-ABS-KEY (child*) AND TITLE-ABS-KEY (educat*) AND TITLE-ABS-KEY (growth))	(78)
84.	(TITLE-ABS-KEY ("neurodevelopmental disorder") AND TITLE-ABS-KEY (child*) AND TITLE-ABS-KEY (educat*) AND TITLE-ABS-KEY (health))	(373)
85.	(TITLE-ABS-KEY ("neurodevelopmental disorder") AND TITLE-ABS-KEY (child*) AND TITLE-ABS-KEY (feed*) AND TITLE-ABS-KEY (nutri*))	(64)

SL	Search details	No of articles
86.	(TITLE-ABS-KEY ("neurodevelopmental disorder") AND TITLE-ABS-KEY (child*) AND TITLE-ABS-KEY (feed*) AND TITLE-ABS-KEY (malnutri*))	(10)
87.	(TITLE-ABS-KEY ("neurodevelopmental disorder") AND TITLE-ABS-KEY (child*) AND TITLE-ABS-KEY (feed*) AND TITLE-ABS-KEY (growth))	(81)
88.	(TITLE-ABS-KEY ("neurodevelopmental disorder") AND TITLE-ABS-KEY (child*) AND TITLE-ABS-KEY (feed*) AND TITLE-ABS-KEY (health))	(80)
89.	(TITLE-ABS-KEY ("neurodevelopmental disorder") AND TITLE-ABS-KEY (child*) AND TITLE-ABS-KEY (surg*) AND TITLE-ABS-KEY (nutri*))	(10)
90.	(TITLE-ABS-KEY ("neurodevelopmental disorder") AND TITLE-ABS-KEY (child*) AND TITLE-ABS-KEY (surg*) AND TITLE-ABS-KEY (malnutri*))	(1)
91.	(TITLE-ABS-KEY ("neurodevelopmental disorder") AND TITLE-ABS-KEY (child*) AND TITLE-ABS-KEY (surg*) AND TITLE-ABS-KEY (growth))	(37)
92.	(TITLE-ABS-KEY ("neurodevelopmental disorder") AND TITLE-ABS-KEY (child*) AND TITLE-ABS-KEY (surg*) AND TITLE-ABS-KEY (health))	(46)
93.	(TITLE-ABS-KEY ("neurodevelopmental disorder") AND TITLE-ABS-KEY (child*) AND TITLE-ABS-KEY (techn*) AND TITLE-ABS-KEY (nutri*))	(14)
94.	(TITLE-ABS-KEY ("neurodevelopmental disorder") AND TITLE-ABS-KEY (child*) AND TITLE-ABS-KEY (techn*) AND TITLE-ABS-KEY (malnutri*))	(4)
95.	(TITLE-ABS-KEY ("neurodevelopmental disorder") AND TITLE-ABS-KEY (child*) AND TITLE-ABS-KEY (techn*) AND TITLE-ABS-KEY (growth))	(66)
96.	(TITLE-ABS-KEY ("neurodevelopmental disorder") AND TITLE-ABS-KEY (child*) AND TITLE-ABS-KEY (techn*) AND TITLE-ABS-KEY (health))	(112)
97.	(TITLE-ABS-KEY ("neurodevelopmental disorder") AND TITLE-ABS-KEY (child*) AND TITLE-ABS-KEY (train*) AND TITLE-ABS-KEY (nutri*))	(7)
98.	(TITLE-ABS-KEY ("neurodevelopmental disorder") AND TITLE-ABS-KEY (child*) AND TITLE-ABS-KEY (train*) AND TITLE-ABS-KEY (malnutri*))	(0)
99.	(TITLE-ABS-KEY ("neurodevelopmental disorder") AND TITLE-ABS-KEY (child*) AND TITLE-ABS-KEY (train*) AND TITLE-ABS-KEY (growth))	(17)
100.	(TITLE-ABS-KEY ("neurodevelopmental disorder") AND TITLE-ABS-KEY (child*) AND TITLE-ABS-KEY (train*) AND TITLE-ABS-KEY (health))	(134)
101.	(TITLE-ABS-KEY ("neurodevelopmental disorder") AND TITLE-ABS-KEY (adolescent*) AND TITLE-ABS-KEY (educat*) AND TITLE-ABS-KEY (nutri*))	(6)
102.	(TITLE-ABS-KEY ("neurodevelopmental disorder") AND TITLE-ABS-KEY (adolescent*) AND TITLE-ABS-KEY (educat*) AND TITLE-ABS-KEY (malnutri*))	(3)
103.	(TITLE-ABS-KEY ("neurodevelopmental disorder") AND TITLE-ABS-KEY (adolescent*) AND TITLE-ABS-KEY (educat*) AND TITLE-ABS-KEY (growth))	(27)
104.	(TITLE-ABS-KEY ("neurodevelopmental disorder") AND TITLE-ABS-KEY (adolescent*) AND TITLE-ABS-KEY (educat*) AND TITLE-ABS-KEY (health))	(160)
105.	(TITLE-ABS-KEY ("neurodevelopmental disorder") AND TITLE-ABS-KEY (adolescent*) AND TITLE-ABS-KEY (feed*) AND TITLE-ABS-KEY (nutri*))	(11)
106.	(TITLE-ABS-KEY ("neurodevelopmental disorder") AND TITLE-ABS-KEY (adolescent*) AND TITLE-ABS-KEY (feed*) AND TITLE-ABS-KEY (malnutri*))	(2)
107.	(TITLE-ABS-KEY ("neurodevelopmental disorder") AND TITLE-ABS-KEY (adolescent*) AND TITLE-ABS-KEY (feed*) AND TITLE-ABS-KEY (growth))	(19)

SL	Search details	No of articles
108.	(TITLE-ABS-KEY ("neurodevelopmental disorder") AND TITLE-ABS-KEY (adolescent*) AND TITLE-ABS-KEY (feed*) AND TITLE-ABS-KEY (health))	(30)
109.	(TITLE-ABS-KEY ("neurodevelopmental disorder") AND TITLE-ABS-KEY (adolescent*) AND TITLE-ABS-KEY (surg*) AND TITLE-ABS-KEY (nutri*))	(5)
110.	(TITLE-ABS-KEY ("neurodevelopmental disorder") AND TITLE-ABS-KEY (adolescent*) AND TITLE-ABS-KEY (surg*) AND TITLE-ABS-KEY (malnutri*))	(1)
111.	(TITLE-ABS-KEY ("neurodevelopmental disorder") AND TITLE-ABS-KEY (adolescent*) AND TITLE-ABS-KEY (surg*) AND TITLE-ABS-KEY (growth))	(11)
112.	(TITLE-ABS-KEY ("neurodevelopmental disorder") AND TITLE-ABS-KEY (adolescent*) AND TITLE-ABS-KEY (surg*) AND TITLE-ABS-KEY (health))	(18)
113.	(TITLE-ABS-KEY ("neurodevelopmental disorder") AND TITLE-ABS-KEY (adolescent*) AND TITLE-ABS-KEY (techn*) AND TITLE-ABS-KEY (nutri*))	(3)
114.	(TITLE-ABS-KEY ("neurodevelopmental disorder") AND TITLE-ABS-KEY (adolescent*) AND TITLE-ABS-KEY (techn*) AND TITLE-ABS-KEY (malnutri*))	(0)
115.	(TITLE-ABS-KEY ("neurodevelopmental disorder") AND TITLE-ABS-KEY (adolescent*) AND TITLE-ABS-KEY (techn*) AND TITLE-ABS-KEY (growth))	(23)
116.	(TITLE-ABS-KEY ("neurodevelopmental disorder") AND TITLE-ABS-KEY (adolescent*) AND TITLE-ABS-KEY (techn*) AND TITLE-ABS-KEY (health))	(41)
117.	(TITLE-ABS-KEY ("neurodevelopmental disorder") AND TITLE-ABS-KEY (adolescent*) AND TITLE-ABS-KEY (train*) AND TITLE-ABS-KEY (nutri*))	(1)
118.	(TITLE-ABS-KEY ("neurodevelopmental disorder") AND TITLE-ABS-KEY (adolescent*) AND TITLE-ABS-KEY (train*) AND TITLE-ABS-KEY (malnutri*))	(0)
119.	(TITLE-ABS-KEY ("neurodevelopmental disorder") AND TITLE-ABS-KEY (adolescent*) AND TITLE-ABS-KEY (train*) AND TITLE-ABS-KEY (growth))	(5)
120.	(TITLE-ABS-KEY ("neurodevelopmental disorder") AND TITLE-ABS-KEY (adolescent*) AND TITLE-ABS-KEY (train*) AND TITLE-ABS-KEY (health))	(52)
121.	(TITLE-ABS-KEY ("neurodevelopmental disorder") AND TITLE-ABS-KEY (infant) AND TITLE-ABS-KEY (educat*) AND TITLE-ABS-KEY (nutri*))	(19)
122.	(TITLE-ABS-KEY ("neurodevelopmental disorder") AND TITLE-ABS-KEY (infant) AND TITLE-ABS-KEY (educat*) AND TITLE-ABS-KEY (malnutri*))	(2)
123.	(TITLE-ABS-KEY ("neurodevelopmental disorder") AND TITLE-ABS-KEY (infant) AND TITLE-ABS-KEY (educat*) AND TITLE-ABS-KEY (growth))	(41)
124.	(TITLE-ABS-KEY ("neurodevelopmental disorder") AND TITLE-ABS-KEY (infant) AND TITLE-ABS-KEY (educat*) AND TITLE-ABS-KEY (health))	(92)
125.	(TITLE-ABS-KEY ("neurodevelopmental disorder") AND TITLE-ABS-KEY (infant) AND TITLE-ABS-KEY (feed*) AND TITLE-ABS-KEY (nutri*))	(52)
126.	(TITLE-ABS-KEY ("neurodevelopmental disorder") AND TITLE-ABS-KEY (infant) AND TITLE-ABS-KEY (feed*) AND TITLE-ABS-KEY (malnutri*))	(5)
127.	(TITLE-ABS-KEY ("neurodevelopmental disorder") AND TITLE-ABS-KEY (infant) AND TITLE-ABS-KEY (feed*) AND TITLE-ABS-KEY (growth))	(60)
128.	(TITLE-ABS-KEY ("neurodevelopmental disorder") AND TITLE-ABS-KEY (infant) AND TITLE-ABS-KEY (feed*) AND TITLE-ABS-KEY (health))	(47)
129.	(TITLE-ABS-KEY ("neurodevelopmental disorder") AND TITLE-ABS-KEY (infant) AND TITLE-ABS-KEY (surg*) AND TITLE-ABS-KEY (nutri*))	(10)

SL	Search details	No of articles
130.	(TITLE-ABS-KEY ("neurodevelopmental disorder") AND TITLE-ABS-KEY (infant) AND TITLE-ABS-KEY (surg*) AND TITLE-ABS-KEY (malnutri*))	(1)
131.	(TITLE-ABS-KEY ("neurodevelopmental disorder") AND TITLE-ABS-KEY (infant) AND TITLE-ABS-KEY (surg*) AND TITLE-ABS-KEY (growth))	(30)
132.	(TITLE-ABS-KEY ("neurodevelopmental disorder") AND TITLE-ABS-KEY (infant) AND TITLE-ABS-KEY (surg*) AND TITLE-ABS-KEY (health))	(25)
133.	(TITLE-ABS-KEY ("neurodevelopmental disorder") AND TITLE-ABS-KEY (infant) AND TITLE-ABS-KEY (techn*) AND TITLE-ABS-KEY (nutri*))	(9)
134.	(TITLE-ABS-KEY ("neurodevelopmental disorder") AND TITLE-ABS-KEY (infant) AND TITLE-ABS-KEY (techn*) AND TITLE-ABS-KEY (malnutri*))	(2)
135.	(TITLE-ABS-KEY ("neurodevelopmental disorder") AND TITLE-ABS-KEY (infant) AND TITLE-ABS-KEY (techn*) AND TITLE-ABS-KEY (growth))	(36)
136.	(TITLE-ABS-KEY ("neurodevelopmental disorder") AND TITLE-ABS-KEY (infant) AND TITLE-ABS-KEY (techn*) AND TITLE-ABS-KEY (health))	(27)
137.	(TITLE-ABS-KEY ("neurodevelopmental disorder") AND TITLE-ABS-KEY (infant) AND TITLE-ABS-KEY (train*) AND TITLE-ABS-KEY (nutri*))	(2)
138.	(TITLE-ABS-KEY ("neurodevelopmental disorder") AND TITLE-ABS-KEY (infant) AND TITLE-ABS-KEY (train*) AND TITLE-ABS-KEY (malnutri*))	(0)
139.	(TITLE-ABS-KEY ("neurodevelopmental disorder") AND TITLE-ABS-KEY (infant) AND TITLE-ABS-KEY (train*) AND TITLE-ABS-KEY (growth))	(8)
140.	(TITLE-ABS-KEY ("neurodevelopmental disorder") AND TITLE-ABS-KEY (infant) AND TITLE-ABS-KEY (train*) AND TITLE-ABS-KEY (health))	(23)
141.	(TITLE-ABS-KEY ("neurodevelopmental disorder") AND TITLE-ABS-KEY (toddler) AND TITLE-ABS-KEY (educat*) AND TITLE-ABS-KEY (nutri*))	(4)
142.	(TITLE-ABS-KEY ("neurodevelopmental disorder") AND TITLE-ABS-KEY (toddler) AND TITLE-ABS-KEY (educat*) AND TITLE-ABS-KEY (malnutri*))	(2)
143.	(TITLE-ABS-KEY ("neurodevelopmental disorder") AND TITLE-ABS-KEY (toddler) AND TITLE-ABS-KEY (educat*) AND TITLE-ABS-KEY (growth))	(7)
144.	(TITLE-ABS-KEY ("neurodevelopmental disorder") AND TITLE-ABS-KEY (toddler) AND TITLE-ABS-KEY (educat*) AND TITLE-ABS-KEY (health))	(13)
145.	(TITLE-ABS-KEY ("neurodevelopmental disorder") AND TITLE-ABS-KEY (toddler) AND TITLE-ABS-KEY (feed*) AND TITLE-ABS-KEY (nutri*))	(2)
146.	(TITLE-ABS-KEY ("neurodevelopmental disorder") AND TITLE-ABS-KEY (toddler) AND TITLE-ABS-KEY (feed*) AND TITLE-ABS-KEY (malnutri*))	(0)
147.	(TITLE-ABS-KEY ("neurodevelopmental disorder") AND TITLE-ABS-KEY (toddler) AND TITLE-ABS-KEY (feed*) AND TITLE-ABS-KEY (growth))	(3)
148.	(TITLE-ABS-KEY ("neurodevelopmental disorder") AND TITLE-ABS-KEY (toddler) AND TITLE-ABS-KEY (feed*) AND TITLE-ABS-KEY (health))	(1)
149.	(TITLE-ABS-KEY ("neurodevelopmental disorder") AND TITLE-ABS-KEY (toddler) AND TITLE-ABS-KEY (surg*) AND TITLE-ABS-KEY (nutri*))	(0)
150.	(TITLE-ABS-KEY ("neurodevelopmental disorder") AND TITLE-ABS-KEY (toddler) AND TITLE-ABS-KEY (surg*) AND TITLE-ABS-KEY (malnutri*))	(0)
151.	(TITLE-ABS-KEY ("neurodevelopmental disorder") AND TITLE-ABS-KEY (toddler) AND TITLE-ABS-KEY (surg*) AND TITLE-ABS-KEY (growth))	(2)

SL	Search details	No of articles
152.	(TITLE-ABS-KEY ("neurodevelopmental disorder") AND TITLE-ABS-KEY (toddler) AND TITLE-ABS-KEY (surg*) AND TITLE-ABS-KEY (health))	(1)
153.	(TITLE-ABS-KEY ("neurodevelopmental disorder") AND TITLE-ABS-KEY (toddler) AND TITLE-ABS-KEY (techn*) AND TITLE-ABS-KEY (nutri*))	(0)
154.	(TITLE-ABS-KEY ("neurodevelopmental disorder") AND TITLE-ABS-KEY (toddler) AND TITLE-ABS-KEY (techn*) AND TITLE-ABS-KEY (malnutri*))	(0)
155.	(TITLE-ABS-KEY ("neurodevelopmental disorder") AND TITLE-ABS-KEY (toddler) AND TITLE-ABS-KEY (techn*) AND TITLE-ABS-KEY (growth))	(1)
156.	(TITLE-ABS-KEY ("neurodevelopmental disorder") AND TITLE-ABS-KEY (toddler) AND TITLE-ABS-KEY (techn*) AND TITLE-ABS-KEY (health))	(2)
157.	(TITLE-ABS-KEY ("neurodevelopmental disorder") AND TITLE-ABS-KEY (toddler) AND TITLE-ABS-KEY (train*) AND TITLE-ABS-KEY (nutri*))	(1)
158.	(TITLE-ABS-KEY ("neurodevelopmental disorder") AND TITLE-ABS-KEY (toddler) AND TITLE-ABS-KEY (train*) AND TITLE-ABS-KEY (malnutri*))	(0)
159.	(TITLE-ABS-KEY ("neurodevelopmental disorder") AND TITLE-ABS-KEY (toddler) AND TITLE-ABS-KEY (train*) AND TITLE-ABS-KEY (growth))	(2)
160.	(TITLE-ABS-KEY ("neurodevelopmental disorder") AND TITLE-ABS-KEY (toddler) AND TITLE-ABS-KEY (train*) AND TITLE-ABS-KEY (health))	(7)
161.	(TITLE-ABS-KEY (spastic*) AND TITLE-ABS-KEY (child*) AND TITLE-ABS-KEY (educat*) AND TITLE-ABS-KEY (nutri*))	(19)
162.	(TITLE-ABS-KEY (spastic*) AND TITLE-ABS-KEY (child*) AND TITLE-ABS-KEY (educat*) AND TITLE-ABS-KEY (malnutri*))	(11)
163.	(TITLE-ABS-KEY (spastic*) AND TITLE-ABS-KEY (child*) AND TITLE-ABS-KEY (educat*) AND TITLE-ABS-KEY (growth))	(14)
164.	(TITLE-ABS-KEY (spastic*) AND TITLE-ABS-KEY (child*) AND TITLE-ABS-KEY (educat*) AND TITLE-ABS-KEY (health))	(118)
165.	(TITLE-ABS-KEY (spastic*) AND TITLE-ABS-KEY (child*) AND TITLE-ABS-KEY (feed*) AND TITLE-ABS-KEY (nutri*))	(91)
166.	(TITLE-ABS-KEY (spastic*) AND TITLE-ABS-KEY (child*) AND TITLE-ABS-KEY (feed*) AND TITLE-ABS-KEY (malnutri*))	(32)
167.	(TITLE-ABS-KEY (spastic*) AND TITLE-ABS-KEY (child*) AND TITLE-ABS-KEY (feed*) AND TITLE-ABS-KEY (growth))	(66)
168.	(TITLE-ABS-KEY (spastic*) AND TITLE-ABS-KEY (child*) AND TITLE-ABS-KEY (feed*) AND TITLE-ABS-KEY (health))	(82)
169.	(TITLE-ABS-KEY (spastic*) AND TITLE-ABS-KEY (child*) AND TITLE-ABS-KEY (surg*) AND TITLE-ABS-KEY (nutri*))	(39)
170.	(TITLE-ABS-KEY (spastic*) AND TITLE-ABS-KEY (child*) AND TITLE-ABS-KEY (surg*) AND TITLE-ABS-KEY (malnutri*))	(9)
171.	(TITLE-ABS-KEY (spastic*) AND TITLE-ABS-KEY (child*) AND TITLE-ABS-KEY (surg*) AND TITLE-ABS-KEY (growth))	(137)
172.	(TITLE-ABS-KEY (spastic*) AND TITLE-ABS-KEY (child*) AND TITLE-ABS-KEY (surg*) AND TITLE-ABS-KEY (health))	(194)
173.	(TITLE-ABS-KEY (spastic*) AND TITLE-ABS-KEY (child*) AND TITLE-ABS-KEY (techn*) AND TITLE-ABS-KEY (nutri*))	(24)

SL	Search details	No of articles
174.	(TITLE-ABS-KEY (spastic*) AND TITLE-ABS-KEY (child*) AND TITLE-ABS-KEY (techn*) AND TITLE-ABS-KEY (malnutri*))	(5)
175.	(TITLE-ABS-KEY (spastic*) AND TITLE-ABS-KEY (child*) AND TITLE-ABS-KEY (techn*) AND TITLE-ABS-KEY (growth))	(83)
176.	(TITLE-ABS-KEY (spastic*) AND TITLE-ABS-KEY (child*) AND TITLE-ABS-KEY (techn*) AND TITLE-ABS-KEY (health))	(125)
177.	(TITLE-ABS-KEY (spastic*) AND TITLE-ABS-KEY (child*) AND TITLE-ABS-KEY (train*) AND TITLE-ABS-KEY (nutri*))	(12)
178.	(TITLE-ABS-KEY (spastic*) AND TITLE-ABS-KEY (child*) AND TITLE-ABS-KEY (train*) AND TITLE-ABS-KEY (malnutri*))	(4)
179.	(TITLE-ABS-KEY (spastic*) AND TITLE-ABS-KEY (child*) AND TITLE-ABS-KEY (train*) AND TITLE-ABS-KEY (growth))	(27)
180.	(TITLE-ABS-KEY (spastic*) AND TITLE-ABS-KEY (child*) AND TITLE-ABS-KEY (train*) AND TITLE-ABS-KEY (health))	(115)
181.	(TITLE-ABS-KEY (spastic*) AND TITLE-ABS-KEY (adolescent*) AND TITLE-ABS-KEY (educat*) AND TITLE-ABS-KEY (nutri*))	(8)
182.	(TITLE-ABS-KEY (spastic*) AND TITLE-ABS-KEY (adolescent*) AND TITLE-ABS-KEY (educat*) AND TITLE-ABS-KEY (malnutri*))	(3)
183.	(TITLE-ABS-KEY (spastic*) AND TITLE-ABS-KEY (adolescent*) AND TITLE-ABS-KEY (educat*) AND TITLE-ABS-KEY (growth))	(2)
184.	(TITLE-ABS-KEY (spastic*) AND TITLE-ABS-KEY (adolescent*) AND TITLE-ABS-KEY (educat*) AND TITLE-ABS-KEY (health))	(44)
185.	(TITLE-ABS-KEY (spastic*) AND TITLE-ABS-KEY (adolescent*) AND TITLE-ABS-KEY (feed*) AND TITLE-ABS-KEY (nutri*))	(39)
186.	(TITLE-ABS-KEY (spastic*) AND TITLE-ABS-KEY (adolescent*) AND TITLE-ABS-KEY (feed*) AND TITLE-ABS-KEY (malnutri*))	(13)
187.	(TITLE-ABS-KEY (spastic*) AND TITLE-ABS-KEY (adolescent*) AND TITLE-ABS-KEY (feed*) AND TITLE-ABS-KEY (growth))	(24)
188..	(TITLE-ABS-KEY (spastic*) AND TITLE-ABS-KEY (adolescent*) AND TITLE-ABS-KEY (feed*) AND TITLE-ABS-KEY (health))	(32)
189.	(TITLE-ABS-KEY (spastic*) AND TITLE-ABS-KEY (infant) AND TITLE-ABS-KEY (educat*) AND TITLE-ABS-KEY (nutri*))	(5)
190.	(TITLE-ABS-KEY (spastic*) AND TITLE-ABS-KEY (infant) AND TITLE-ABS-KEY (educat*) AND TITLE-ABS-KEY (malnutri*))	(3)
191.	(TITLE-ABS-KEY (spastic*) AND TITLE-ABS-KEY (infant) AND TITLE-ABS-KEY (educat*) AND TITLE-ABS-KEY (growth))	(2)
192.	(TITLE-ABS-KEY (spastic*) AND TITLE-ABS-KEY (infant) AND TITLE-ABS-KEY (educat*) AND TITLE-ABS-KEY (health))	(33)
193.	(TITLE-ABS-KEY (spastic*) AND TITLE-ABS-KEY (infant) AND TITLE-ABS-KEY (feed*) AND TITLE-ABS-KEY (nutri*))	(35)
194.	(TITLE-ABS-KEY (spastic*) AND TITLE-ABS-KEY (infant) AND TITLE-ABS-KEY (feed*) AND TITLE-ABS-KEY (growth))	(37)
195.	(TITLE-ABS-KEY (spastic*) AND TITLE-ABS-KEY (infant) AND TITLE-ABS-KEY (feed*) AND TITLE-ABS-KEY (health))	(27)

SL	Search details	No of articles
196.	(TITLE-ABS-KEY (spastic*) AND TITLE-ABS-KEY (infant) AND TITLE-ABS-KEY (surg*) AND TITLE-ABS-KEY (nutri*))	(13)
197.	(TITLE-ABS-KEY (spastic*) AND TITLE-ABS-KEY (infant) AND TITLE-ABS-KEY (surg*) AND TITLE-ABS-KEY (malnutri*))	(3)
198.	(TITLE-ABS-KEY (spastic*) AND TITLE-ABS-KEY (infant) AND TITLE-ABS-KEY (surg*) AND TITLE-ABS-KEY (growth))	(36)
199.	(TITLE-ABS-KEY (spastic*) AND TITLE-ABS-KEY (infant) AND TITLE-ABS-KEY (surg*) AND TITLE-ABS-KEY (health))	(30)
200.	(TITLE-ABS-KEY (spastic*) AND TITLE-ABS-KEY (infant) AND TITLE-ABS-KEY (techn*) AND TITLE-ABS-KEY (nutri*))	(6)
201.	(TITLE-ABS-KEY (spastic*) AND TITLE-ABS-KEY (infant) AND TITLE-ABS-KEY (techn*) AND TITLE-ABS-KEY (malnutri*))	(1)
202.	(TITLE-ABS-KEY (spastic*) AND TITLE-ABS-KEY (infant) AND TITLE-ABS-KEY (techn*) AND TITLE-ABS-KEY (growth))	(24)
203.	(TITLE-ABS-KEY (spastic*) AND TITLE-ABS-KEY (infant) AND TITLE-ABS-KEY (techn*) AND TITLE-ABS-KEY (health))	(17)
204.	(TITLE-ABS-KEY (spastic*) AND TITLE-ABS-KEY (infant) AND TITLE-ABS-KEY (train*) AND TITLE-ABS-KEY (nutri*))	(1)
205.	(TITLE-ABS-KEY (spastic*) AND TITLE-ABS-KEY (infant) AND TITLE-ABS-KEY (train*) AND TITLE-ABS-KEY (malnutri*))	(1)
206.	(TITLE-ABS-KEY (spastic*) AND TITLE-ABS-KEY (infant) AND TITLE-ABS-KEY (train*) AND TITLE-ABS-KEY (growth))	(3)
207.	(TITLE-ABS-KEY (spastic*) AND TITLE-ABS-KEY (infant) AND TITLE-ABS-KEY (train*) AND TITLE-ABS-KEY (health))	(8)
208.	(TITLE-ABS-KEY (spastic*) AND TITLE-ABS-KEY (toddler) AND TITLE-ABS-KEY (educat*) AND TITLE-ABS-KEY (nutri*))	(0)
209.	(TITLE-ABS-KEY (spastic*) AND TITLE-ABS-KEY (toddler) AND TITLE-ABS-KEY (educat*) AND TITLE-ABS-KEY (malnutri*))	(0)
210.	(TITLE-ABS-KEY (spastic*) AND TITLE-ABS-KEY (toddler) AND TITLE-ABS-KEY (educat*) AND TITLE-ABS-KEY (growth))	(0)
211.	(TITLE-ABS-KEY (spastic*) AND TITLE-ABS-KEY (toddler) AND TITLE-ABS-KEY (educat*) AND TITLE-ABS-KEY (health))	(0)
212.	(TITLE-ABS-KEY (spastic*) AND TITLE-ABS-KEY (toddler) AND TITLE-ABS-KEY (feed*) AND TITLE-ABS-KEY (nutri*))	(1)
213.	(TITLE-ABS-KEY (spastic*) AND TITLE-ABS-KEY (toddler) AND TITLE-ABS-KEY (feed*) AND TITLE-ABS-KEY (malnutri*))	(0)
214.	(TITLE-ABS-KEY (spastic*) AND TITLE-ABS-KEY (toddler) AND TITLE-ABS-KEY (feed*) AND TITLE-ABS-KEY (growth))	(0)
215.	(TITLE-ABS-KEY (spastic*) AND TITLE-ABS-KEY (toddler) AND TITLE-ABS-KEY (feed*) AND TITLE-ABS-KEY (health))	(0)
216.	(TITLE-ABS-KEY (spastic*) AND TITLE-ABS-KEY (toddler) AND TITLE-ABS-KEY (surg*) AND TITLE-ABS-KEY (nutri*))	(0)
217.	(TITLE-ABS-KEY (spastic*) AND TITLE-ABS-KEY (toddler) AND TITLE-ABS-KEY (surg*) AND TITLE-ABS-KEY (malnutri*))	(0)

SL	Search details	No of articles
218.	(TITLE-ABS-KEY (spastic*) AND TITLE-ABS-KEY (toddler) AND TITLE-ABS-KEY (surg*) AND TITLE-ABS-KEY (growth))	(0)
219.	(TITLE-ABS-KEY (spastic*) AND TITLE-ABS-KEY (toddler) AND TITLE-ABS-KEY (surg*) AND TITLE-ABS-KEY (health))	(0)
220.	(TITLE-ABS-KEY (spastic*) AND TITLE-ABS-KEY (toddler) AND TITLE-ABS-KEY (techn*) AND TITLE-ABS-KEY (nutri*))	(0)
221.	(TITLE-ABS-KEY (spastic*) AND TITLE-ABS-KEY (toddler) AND TITLE-ABS-KEY (techn*) AND TITLE-ABS-KEY (malnutri*))	(0)
222.	(TITLE-ABS-KEY (spastic*) AND TITLE-ABS-KEY (toddler) AND TITLE-ABS-KEY (techn*) AND TITLE-ABS-KEY (growth))	(0)
223.	(TITLE-ABS-KEY (spastic*) AND TITLE-ABS-KEY (toddler) AND TITLE-ABS-KEY (techn*) AND TITLE-ABS-KEY (health))	(0)
224.	(TITLE-ABS-KEY (spastic*) AND TITLE-ABS-KEY (toddler) AND TITLE-ABS-KEY (train*) AND TITLE-ABS-KEY (nutri*))	(0)
225.	(TITLE-ABS-KEY (spastic*) AND TITLE-ABS-KEY (toddler) AND TITLE-ABS-KEY (train*) AND TITLE-ABS-KEY (malnutri*))	(0)
226.	(TITLE-ABS-KEY (spastic*) AND TITLE-ABS-KEY (toddler) AND TITLE-ABS-KEY (train*) AND TITLE-ABS-KEY (growth))	(1)
227.	(TITLE-ABS-KEY (spastic*) AND TITLE-ABS-KEY (toddler) AND TITLE-ABS-KEY (train*) AND TITLE-ABS-KEY (health))	(1)
228.	(TITLE-ABS-KEY (dyskine*) AND TITLE-ABS-KEY (child*) AND TITLE-ABS-KEY (surg*) AND TITLE-ABS-KEY (health))	(50)
229.	(TITLE-ABS-KEY (dyskine*) AND TITLE-ABS-KEY (child*) AND TITLE-ABS-KEY (techn*) AND TITLE-ABS-KEY (nutri*))	(7)
230.	(TITLE-ABS-KEY (dyskine*) AND TITLE-ABS-KEY (child*) AND TITLE-ABS-KEY (techn*) AND TITLE-ABS-KEY (malnutri*))	(1)
231.	(TITLE-ABS-KEY (dyskine*) AND TITLE-ABS-KEY (child*) AND TITLE-ABS-KEY (techn*) AND TITLE-ABS-KEY (growth))	(8)
232..	(TITLE-ABS-KEY (dyskine*) AND TITLE-ABS-KEY (child*) AND TITLE-ABS-KEY (techn*) AND TITLE-ABS-KEY (health))	(39)
233.	(TITLE-ABS-KEY (dyskine*) AND TITLE-ABS-KEY (child*) AND TITLE-ABS-KEY (train*) AND TITLE-ABS-KEY (nutri*))	(2)
234.	(TITLE-ABS-KEY (dyskine*) AND TITLE-ABS-KEY (child*) AND TITLE-ABS-KEY (train*) AND TITLE-ABS-KEY (malnutri*))	(1)
235.	(TITLE-ABS-KEY (dyskine*) AND TITLE-ABS-KEY (child*) AND TITLE-ABS-KEY (train*) AND TITLE-ABS-KEY (growth))	(5)
236.	(TITLE-ABS-KEY (dyskine*) AND TITLE-ABS-KEY (child*) AND TITLE-ABS-KEY (train*) AND TITLE-ABS-KEY (health))	(18)
237.	(TITLE-ABS-KEY (dyskine*) AND TITLE-ABS-KEY (adolescent*) AND TITLE-ABS-KEY (educat*) AND TITLE-ABS-KEY (nutri*))	(1)
238.	(TITLE-ABS-KEY (dyskine*) AND TITLE-ABS-KEY (adolescent*) AND TITLE-ABS-KEY (educat*) AND TITLE-ABS-KEY (malnutri*))	(0)
239.	(TITLE-ABS-KEY (dyskine*) AND TITLE-ABS-KEY (adolescent*) AND TITLE-ABS-KEY (educat*) AND TITLE-ABS-KEY (growth))	(0)

SL	Search details	No of articles
240.	(TITLE-ABS-KEY (dyskine*) AND TITLE-ABS-KEY (adolescent*) AND TITLE-ABS-KEY (educat*) AND TITLE-ABS-KEY (health))	(33)
241.	(TITLE-ABS-KEY (dyskine*) AND TITLE-ABS-KEY (adolescent*) AND TITLE-ABS-KEY (feed*) AND TITLE-ABS-KEY (nutri*))	(4)
242.	(TITLE-ABS-KEY (dyskine*) AND TITLE-ABS-KEY (adolescent*) AND TITLE-ABS-KEY (feed*) AND TITLE-ABS-KEY (malnutri*))	(0)
243.	(TITLE-ABS-KEY (dyskine*) AND TITLE-ABS-KEY (adolescent*) AND TITLE-ABS-KEY (feed*) AND TITLE-ABS-KEY (growth))	(6)
244.	(TITLE-ABS-KEY (dyskine*) AND TITLE-ABS-KEY (adolescent*) AND TITLE-ABS-KEY (feed*) AND TITLE-ABS-KEY (health))	(4)
245.	(TITLE-ABS-KEY (dyskine*) AND TITLE-ABS-KEY (adolescent*) AND TITLE-ABS-KEY (surg*) AND TITLE-ABS-KEY (nutri*))	(8)
246.	(TITLE-ABS-KEY (dyskine*) AND TITLE-ABS-KEY (adolescent*) AND TITLE-ABS-KEY (surg*) AND TITLE-ABS-KEY (malnutri*))	(0)
247.	(TITLE-ABS-KEY (dyskine*) AND TITLE-ABS-KEY (adolescent*) AND TITLE-ABS-KEY (surg*) AND TITLE-ABS-KEY (growth))	(6)
248.	(TITLE-ABS-KEY (dyskine*) AND TITLE-ABS-KEY (adolescent*) AND TITLE-ABS-KEY (surg*) AND TITLE-ABS-KEY (health))	(39)
249.	(TITLE-ABS-KEY (dyskine*) AND TITLE-ABS-KEY (adolescent*) AND TITLE-ABS-KEY (techn*) AND TITLE-ABS-KEY (nutri*))	(3)
250.	(TITLE-ABS-KEY (dyskine*) AND TITLE-ABS-KEY (adolescent*) AND TITLE-ABS-KEY (techn*) AND TITLE-ABS-KEY (malnutri*))	(0)
251.	(TITLE-ABS-KEY (dyskine*) AND TITLE-ABS-KEY (adolescent*) AND TITLE-ABS-KEY (techn*) AND TITLE-ABS-KEY (growth))	(4)
252.	(TITLE-ABS-KEY (dyskine*) AND TITLE-ABS-KEY (adolescent*) AND TITLE-ABS-KEY (techn*) AND TITLE-ABS-KEY (health))	(23)
253.	(TITLE-ABS-KEY (dyskine*) AND TITLE-ABS-KEY (adolescent*) AND TITLE-ABS-KEY (train*) AND TITLE-ABS-KEY (nutri*))	(0)
254.	(TITLE-ABS-KEY (dyskine*) AND TITLE-ABS-KEY (adolescent*) AND TITLE-ABS-KEY (train*) AND TITLE-ABS-KEY (malnutri*))	(0)
255.	(TITLE-ABS-KEY (dyskine*) AND TITLE-ABS-KEY (adolescent*) AND TITLE-ABS-KEY (train*) AND TITLE-ABS-KEY (growth))	(4)
256.	(TITLE-ABS-KEY (dyskine*) AND TITLE-ABS-KEY (adolescent*) AND TITLE-ABS-KEY (train*) AND TITLE-ABS-KEY (growth))	(4)
257.	(TITLE-ABS-KEY (dyskine*) AND TITLE-ABS-KEY (adolescent*) AND TITLE-ABS-KEY (train*) AND TITLE-ABS-KEY (health))	(10)
258.	(TITLE-ABS-KEY (dyskine*) AND TITLE-ABS-KEY (infant) AND TITLE-ABS-KEY (educat*) AND TITLE-ABS-KEY (nutri*))	(1)
259.	(TITLE-ABS-KEY (dyskine*) AND TITLE-ABS-KEY (infant) AND TITLE-ABS-KEY (educat*) AND TITLE-ABS-KEY (malnutri*))	(0)
260.	(TITLE-ABS-KEY (dyskine*) AND TITLE-ABS-KEY (infant) AND TITLE-ABS-KEY (educat*) AND TITLE-ABS-KEY (growth))	(2)
261.	(TITLE-ABS-KEY (dyskine*) AND TITLE-ABS-KEY (infant) AND TITLE-ABS-KEY (educat*) AND TITLE-ABS-KEY (health))	(6)

SL	Search details	No of articles
262.	(TITLE-ABS-KEY (dyskine*) AND TITLE-ABS-KEY (infant) AND TITLE-ABS-KEY (feed*) AND TITLE-ABS-KEY (nutri*))	(12)
263.	(TITLE-ABS-KEY (dyskine*) AND TITLE-ABS-KEY (infant) AND TITLE-ABS-KEY (feed*) AND TITLE-ABS-KEY (malnutri*))	(3)
264.	(TITLE-ABS-KEY (dyskine*) AND TITLE-ABS-KEY (infant) AND TITLE-ABS-KEY (feed*) AND TITLE-ABS-KEY (growth))	(12)
265.	(TITLE-ABS-KEY (dyskine*) AND TITLE-ABS-KEY (infant) AND TITLE-ABS-KEY (feed*) AND TITLE-ABS-KEY (health))	(7)
266.	(TITLE-ABS-KEY (dyskine*) AND TITLE-ABS-KEY (infant) AND TITLE-ABS-KEY (surg*) AND TITLE-ABS-KEY (nutri*))	(1)
267.	(TITLE-ABS-KEY (dyskine*) AND TITLE-ABS-KEY (infant) AND TITLE-ABS-KEY (surg*) AND TITLE-ABS-KEY (malnutri*))	(2)
268.	(TITLE-ABS-KEY (dyskine*) AND TITLE-ABS-KEY (infant) AND TITLE-ABS-KEY (surg*) AND TITLE-ABS-KEY (growth))	(2)
269.	(TITLE-ABS-KEY (dyskine*) AND TITLE-ABS-KEY (infant) AND TITLE-ABS-KEY (surg*) AND TITLE-ABS-KEY (health))	(6)
270.	(TITLE-ABS-KEY (dyskine*) AND TITLE-ABS-KEY (infant) AND TITLE-ABS-KEY (techn*) AND TITLE-ABS-KEY (nutri*))	(2)
271.	(TITLE-ABS-KEY (dyskine*) AND TITLE-ABS-KEY (infant) AND TITLE-ABS-KEY (techn*) AND TITLE-ABS-KEY (malnutri*))	(1)
272.	(TITLE-ABS-KEY (dyskine*) AND TITLE-ABS-KEY (infant) AND TITLE-ABS-KEY (techn*) AND TITLE-ABS-KEY (growth))	(1)
273.	(TITLE-ABS-KEY (dyskine*) AND TITLE-ABS-KEY (infant) AND TITLE-ABS-KEY (techn*) AND TITLE-ABS-KEY (health))	(7)
274.	(TITLE-ABS-KEY (dyskine*) AND TITLE-ABS-KEY (infant) AND TITLE-ABS-KEY (train*) AND TITLE-ABS-KEY (nutri*))	(0)
275.	(TITLE-ABS-KEY (dyskine*) AND TITLE-ABS-KEY (infant) AND TITLE-ABS-KEY (train*) AND TITLE-ABS-KEY (malnutri*))	(0)
276.	(TITLE-ABS-KEY (dyskine*) AND TITLE-ABS-KEY (infant) AND TITLE-ABS-KEY (train*) AND TITLE-ABS-KEY (growth))	(1)
277.	(TITLE-ABS-KEY (dyskine*) AND TITLE-ABS-KEY (infant) AND TITLE-ABS-KEY (train*) AND TITLE-ABS-KEY (health))	(2)
278.	(TITLE-ABS-KEY (dyskine*) AND TITLE-ABS-KEY (toddler) AND TITLE-ABS-KEY (educat*) AND TITLE-ABS-KEY (nutri*))	(0)
279.	(TITLE-ABS-KEY (dyskine*) AND TITLE-ABS-KEY (toddler) AND TITLE-ABS-KEY (educat*) AND TITLE-ABS-KEY (malnutri*))	(0)
280.	(TITLE-ABS-KEY (dyskine*) AND TITLE-ABS-KEY (toddler) AND TITLE-ABS-KEY (educat*) AND TITLE-ABS-KEY (growth))	(0)
281.	(TITLE-ABS-KEY (dyskine*) AND TITLE-ABS-KEY (toddler) AND TITLE-ABS-KEY (educat*) AND TITLE-ABS-KEY (health))	(0)
282.	(TITLE-ABS-KEY (dyskine*) AND TITLE-ABS-KEY (toddler) AND TITLE-ABS-KEY (feed*) AND TITLE-ABS-KEY (nutri*))	(0)
283.	(TITLE-ABS-KEY (dyskine*) AND TITLE-ABS-KEY (toddler) AND TITLE-ABS-KEY (feed*) AND TITLE-ABS-KEY (malnutri*))	(0)

SL	Search details	No of articles
284.	(TITLE-ABS-KEY (dyskine*) AND TITLE-ABS-KEY (toddler) AND TITLE-ABS-KEY (feed*) AND TITLE-ABS-KEY (growth))	(0)
285.	(TITLE-ABS-KEY (dyskine*) AND TITLE-ABS-KEY (toddler) AND TITLE-ABS-KEY (feed*) AND TITLE-ABS-KEY (health))	(0)
286.	(TITLE-ABS-KEY (dyskine*) AND TITLE-ABS-KEY (toddler) AND TITLE-ABS-KEY (surg*) AND TITLE-ABS-KEY (nutri*))	(0)
287.	(TITLE-ABS-KEY (dyskine*) AND TITLE-ABS-KEY (toddler) AND TITLE-ABS-KEY (surg*) AND TITLE-ABS-KEY (malnutri*))	(0)
288.	(TITLE-ABS-KEY (dyskine*) AND TITLE-ABS-KEY (toddler) AND TITLE-ABS-KEY (surg*) AND TITLE-ABS-KEY (growth))	(0)
289.	(TITLE-ABS-KEY (dyskine*) AND TITLE-ABS-KEY (toddler) AND TITLE-ABS-KEY (surg*) AND TITLE-ABS-KEY (health))	(0)
290.	(TITLE-ABS-KEY (dyskine*) AND TITLE-ABS-KEY (toddler) AND TITLE-ABS-KEY (techn*) AND TITLE-ABS-KEY (nutri*))	(0)
291.	(TITLE-ABS-KEY (dyskine*) AND TITLE-ABS-KEY (toddler) AND TITLE-ABS-KEY (techn*) AND TITLE-ABS-KEY (malnutri*))	(0)
292.	(TITLE-ABS-KEY (dyskine*) AND TITLE-ABS-KEY (toddler) AND TITLE-ABS-KEY (techn*) AND TITLE-ABS-KEY (growth))	(0)
293.	(TITLE-ABS-KEY (dyskine*) AND TITLE-ABS-KEY (toddler) AND TITLE-ABS-KEY (techn*) AND TITLE-ABS-KEY (health))	(0)
294.	(TITLE-ABS-KEY (dyskine*) AND TITLE-ABS-KEY (toddler) AND TITLE-ABS-KEY (train*) AND TITLE-ABS-KEY (malnutri*))	(0)
295.	(TITLE-ABS-KEY (dyskine*) AND TITLE-ABS-KEY (toddler) AND TITLE-ABS-KEY (train*) AND TITLE-ABS-KEY (growth))	(0)
296.	(TITLE-ABS-KEY (dyskine*) AND TITLE-ABS-KEY (toddler) AND TITLE-ABS-KEY (train*) AND TITLE-ABS-KEY (health))	(0)
297.	(TITLE-ABS-KEY (dyskine*) AND TITLE-ABS-KEY (child*) AND TITLE-ABS-KEY (educat*) AND TITLE-ABS-KEY (nutri*))	(4)
298.	(TITLE-ABS-KEY (dyskine*) AND TITLE-ABS-KEY (child*) AND TITLE-ABS-KEY (educat*) AND TITLE-ABS-KEY (malnutri*))	(0)
299.	(TITLE-ABS-KEY (dyskine*) AND TITLE-ABS-KEY (child*) AND TITLE-ABS-KEY (educat*) AND TITLE-ABS-KEY (growth))	(2)
300.	(TITLE-ABS-KEY (dyskine*) AND TITLE-ABS-KEY (child*) AND TITLE-ABS-KEY (educat*) AND TITLE-ABS-KEY (health))	(47)
301.	(TITLE-ABS-KEY (dyskine*) AND TITLE-ABS-KEY (child*) AND TITLE-ABS-KEY (feed*) AND TITLE-ABS-KEY (nutri*))	(14)
302.	(TITLE-ABS-KEY (dyskine*) AND TITLE-ABS-KEY (child*) AND TITLE-ABS-KEY (feed*) AND TITLE-ABS-KEY (malnutri*))	(6)
303.	(TITLE-ABS-KEY (dyskine*) AND TITLE-ABS-KEY (child*) AND TITLE-ABS-KEY (feed*) AND TITLE-ABS-KEY (growth))	(16)
304.	(TITLE-ABS-KEY (dyskine*) AND TITLE-ABS-KEY (child*) AND TITLE-ABS-KEY (feed*) AND TITLE-ABS-KEY (health))	(19)
305.	(TITLE-ABS-KEY (dyskine*) AND TITLE-ABS-KEY (child*) AND TITLE-ABS-KEY (surg*) AND TITLE-ABS-KEY (nutri*))	(14)

SL	Search details	No of articles
306.	(TITLE-ABS-KEY (dyskine*) AND TITLE-ABS-KEY (child*) AND TITLE-ABS-KEY (surg*) AND TITLE-ABS-KEY (malnutri*))	(2)
307.	(TITLE-ABS-KEY (dyskine*) AND TITLE-ABS-KEY (child*) AND TITLE-ABS-KEY (surg*) AND TITLE-ABS-KEY (growth))	(14)

Table S2: Summary of the study characteristics of the studies excluded after full article review and reason for exclusion

Study details	Reason for exclusion	Country	Settings	Study design	Study period	Study participants	Characteristics of participating children with CP				Intervention provided	Anthropometric measure, Nutritional indicators used	Nutritional status improved following intervention	Any adverse outcome reported
							Number	Age (range, mean (SD), median [IQR])	Sex	GMFCS level				
Jadi et al , 2021 [1]	Conducted in a HIC	USA	Hospital/ Clinic/ Centre - based	Descriptive analytical-retrospective chart review	Apr 2014 – Dec 2017	Children with CP who underwent gastrostomy tube placement	63	Range: 0.5m -17y; Median [IQR]: 2y [0y 8y]	F: 33, M: 30	Not reported	Gastrostomy tube placement	Weight (kg); WAZ	Z scores improved in 81% children at 3 months, 44% at 6 months, and 64% at 12 months.	69.8% participants experienced some complication, n=7 deceased.
Harberfellner et al.,2000 [2]	Conducted in a HIC	Canada	Special school-based	Experimental	Feb 1994 – Feb 1996	Children with quadriplegic CP	20	Range: 4y 2m -13y 1mo Mean ± SD: 8y 4m ± 0y 11m	F: 11, M: 9	Not reported	Intraoral appliance (ISMAR) therapy	Weight (kg) and height (cm); WAZ and HAZ; TSF (mm), triceps centile, SSF (mm), SSF centile	No significant effect of intervention on weight gain and increase in height of participating children	Not reported
Vernon-Roberts et al., 2010 [3]	Conducted in a HIC	United Kingdom (UK)	Hospital/ Clinic/ Centre - based	Descriptive analytical	Not reported	Children with quadriplegic CP	14	Median [IQR]: 2y Range: [10m-11y]	F: 7, M: 7	GMFCS level III-V: All	Gastrostomy tube insertion	Weight (kg), MUAC (cm), UAL (cm), LLL (cm), TSF (mm), SSF (mm); WAZ	Significant improvement in Weight, MUAC, LLL but not in WAZ, UAL, TSF, SSF	n=3 deceased from respiratory infections
Sullivan et al., 2005 [4]	Conducted in a HIC	UK	Hospital/ Clinic/ Centre - based	Descriptive analytical	Dec 1999 to Dec 2002	Children with CP whom a gastrostomy was clinically indicated	57	Range: 5m-17y 3 m, Median: 4y 4m	F: 28, M: 29	GMFCS level V: 44 of 57	Insertion of a gastrostomy tube	Weight (kg), LLL, UAL, MUAC, TSF, SSF; z scores for each measure	z scores improved for all indicators between baseline and 12 months after intervention	Carer reported complications from gastrostomy placement
Henderson et al.,2007 [5]	Conducted in HICs	USA, Canada	Hospital/ Clinic/ Centre - based	Descriptive comparative	Not reported	Children with CP	Residential center: 75; home	Range: 2y-18y, Mean ± SE: 13y ± 6m (residential	F: 32 (residential care), 95	All GMFCS level IV-V	Residential care	Weight (kg), Height (cm), TSF, SSF, UAMA; z	Children at residential care had significantly better z scores for height and TSF	Not reported

Study details	Reason for exclusion	Country	Settings	Study design	Study period	Study participants	Characteristics of participating children with CP				Intervention provided	Anthropometric measure, Nutritional indicators used	Nutritional status improved following intervention	Any adverse outcome reported
							Number	Age (range, mean (SD), median [IQR])	Sex	GMFCS level				
							- living: 205	centre) vs. 9y 6m ± 4m	(home living), M: 43 (residential care), 110 (home living)			scores for each measure	but not for weight, SSF and UAMA	
Campanozzi et al.,2006 [6]	Conducted in a HIC	Italy	Hospital/ Clinic/ Centre - based	Experimental	N/A	Children with CP and malnutrition	14	Range: 1y-14y	F: 3, M: 11	Not reported	Nutritional rehabilitation, increase in calorie intake	Weight (kg), length (cm), W/H, BMI(kg/m²), FFM, FBM, skinfold anthropometry	FFM and FBM improved following intervention among children	Not reported
Kernizan et al.,2020 [7]	Conducted in a HIC; disaggregated data for children with CP were not reported	USA	Hospital/ Clinic/ Centre - based	Descriptive analytical-retrospective chart review	Jan 2013 to Apr 2017	pediatric patient <22 years including children with CP	Total n=35, children with CP n=5	Disaggregated data were not reported for children with CP	Disaggregated data were not reported for children with CP	Not reported	Dietary modification (blenderized tube feedings)	Weight and height percentiles, BMI percentiles	Disaggregated data were not reported for children with CP	Disaggregated data were not reported for children with CP
Dipasquale et al.,2018 [8]	Conducted in a HIC, and disaggregated data for children with CP	Italy	Hospital/ Clinic/ Centre - based	Descriptive analytical-retrospective study	January 2013 to November 2017	Children with neurological impairment including CP	Total n=38, Children with C n=28	Disaggregated data for children with CP were not reported	Disaggregated data for children with CP were not	Disaggregated data for children with CP were not reported	Percutaneous endoscopic gastrostomy placement, Dietary modification (standard energy density	Body weight (z score), BMI (z score), TSF (z score)	Significant improvement in z scores for body weight, BMI and TSF among children with CP (p<0.001 for all)	Disaggregated data for children with CP were not reported

Study details	Reason for exclusion	Country	Settings	Study design	Study period	Study participants	Characteristics of participating children with CP				Intervention provided	Anthropometric measure, Nutritional indicators used	Nutritional status improved following intervention	Any adverse outcome reported
							Number	Age (range, mean (SD), median [IQR])	Sex	GMFCS level				
	were not reported								reported		polymeric formula)			
Clawson et al.,2007 [9]	Conducted in a HIC	Not reported	Hospital/ Clinic/ Centre -based	Experimental	3 years	Children with diplegic CP	8	Range: 1y 6m-4y 8m; Mean ± SD:2y 10m ± 1y 2m	F: 4, M: 4	Not reported	Beckman oral motor exercises, behavior interventions including differential attention, prolonged presentation, parents training on food preparation and calorie boosting	Weight, height, percentiles	Both weight and height improved gradually throughout the intervention period and at discharge.	Not reported
Corwin et al.,1996 [10]	Conducted in a HIC	USA	Hospital/ Clinic/ Centre -based	Descriptive longitudinal study	Not reported	Gastrostomy-fed children including children with CP	Total n=75, children with CP n=37	Disaggregated data for children with CP were not reported	Disaggregated data for children with CP were not reported	Disaggregated data for children with CP were not reported	Gastrostomy placement	Weight, height, height/weight	Significant increase in weight, height and height/weight at 12 months of follow-up (p=0.005, p=0.0001 and p=0.0002 for height, weight and height/weight respectively) and 18 months (p=0.007,p= 0.0001 and p=0.01 for height, weight, height/weight respectively)	Not reported

Study details	Reason for exclusion	Country	Settings	Study design	Study period	Study participants	Characteristics of participating children with CP				Intervention provided	Anthropometric measure, Nutritional indicators used	Nutritional status improved following intervention	Any adverse outcome reported
							Number	Age (range, mean (SD), median [IQR])	Sex	GMFCS level				
Kong et al.,2005 [11]	Conducted in a HIC	Hong Kong	Hospital/ Clinic/ Centre - based	Descriptive comparative	Not reported	Children with quadriplegic CP (tube fed vs. orally fed) and children without CP	n=110, n=48 tube fed, n=62 orally fed	Not reported	F: 54, M: 56	Not reported	Not applicable (comparative study)	Body weight, recumbent length, Right triceps, anterior mid-thigh, and medial calf skinfold thicknesses; WHZ, BMI, HZ	No significant difference in WHZ between tube fed children with CP and children without CP. However, the WHZ was significantly lower among children with CP fed orally than children who had tube feeding or children without CP	Not reported
Rampel GR. Et al,1988 [12]	Conducted in a HIC	USA	Hospital/ Clinic/ Centre - based	Descriptive longitudinal study	July and November 1986	Children feeding with gastrostomies attending the CP clinic	n=57	Range: 9m-23y 4m, mean: 10y 8m	F: 24, M: 33	Not reported	Gastrostomy feeding	Weight, height, weight for height	Improvement in all three measures observed after gastrostomy, however, malnutrition was common in children even after gastrostomy placement.	23% children had major complications following gastrostomy were reported.
Arrowsmit et al.,2010 [13]	Conducted in a HIC	Australia	Hospital/ Clinic/ Centre - based	Experimental	Between 2000 and 2008.	Children with quadriplegic CP	n=21	Range: 4y-18y; Median [IQR]: 8y5m [6y9m 11y10m]	F: 9, M: 12	GMFCS level V: All	Gastrostomy feeding and dietary modification	weight, height, and skinfold thicknesses (triceps, biceps, subscapular, suprailiac), bone mineral content (BMC), total	Significant improvement was observed in median weight and, height, % body fat, TBP, %TBP predicted for height and BMC. However, the changes were not significant for	Not reported

Study details	Reason for exclusion	Country	Settings	Study design	Study period	Study participants	Characteristics of participating children with CP				Intervention provided	Anthropometric measure, Nutritional indicators used	Nutritional status improved following intervention	Any adverse outcome reported
							Number	Age (range, mean (SD), median [IQR])	Sex	GMFCS level				
												body protein (TBP)	height standard deviation score and TBP for age %.	
Mahant et al.,2009 [14]	Conducted in a HIC	Canada	Hospital/ Clinic/ Centre - based	Descriptive longitudinal study	September 2002.	Children with neurological disorder including CP	n=42	Median [IQR]: 1y 7mo [0.6m, 15y 6m]	F: 23, M: 19	GMFCS V: 29/50, GMFCS IV 6/50, GMFCS III 5/50	Image-guided gastrostomy or gastrojejunostomy tube insertion	weight, length and triceps skinfold thickness, WAZ, HAZ	WAZ improved significantly over time ($p<0.01$), no significant changes were observed for HAZ ($p=0.30$). However, TSF improved in most children.	Complications related to the tube insertion were reported.
Pinnington et al.,1999 [15]	Conducted in a HIC, and disaggregated data for children with CP were not reported	UK	Hospital/ Clinic/ Centre - based	Trial employing AB within-subjects design	9 month period	Children with severe neurological disorder	Disaggregated data for children with CP were not reported	Disaggregated data for children with CP were not reported	Disaggregated data for children with CP were not reported	Disaggregated data for children with CP were not reported	Assistive feeding device	Disaggregated data for children with CP were not reported	Disaggregated data for children with CP were not reported	
Gisel G. et al.,2003 [16]	Conducted in a HIC	Not reported	Hospital/ Clinic/ Centre - based	Case series	June 1998 and February 1999	Children with quadriplegic CP	n=3	Range: 1y 6m-3y 5m	F: 3, M: 0	Not reported	Food texture modification, positioning of child to minimize/eliminate aspiration, calorie and nutrient content modification,	Weight and length	All three children had improved sufficient weight, however, catchup growth in length was observed only for children who were tube fed	Not reported

Study details	Reason for exclusion	Country	Settings	Study design	Study period	Study participants	Characteristics of participating children with CP				Intervention provided	Anthropometric measure, Nutritional indicators used	Nutritional status improved following intervention	Any adverse outcome reported
							Number	Age (range, mean (SD), median [IQR])	Sex	GMFCS level				
											training to mothers on oral-motor excercse for their children,			
Brant et al., 1999 [17]	Disaggregated data for children with CP were not reported	Brazil	Hospital/ Clinic/ Centre - based	Case series	August 1996 to August 1997	Children with CP, myopathy, and brain trauma	16	Disaggregated data for children with CP were not reported	Disaggregated data for children with CP were not reported	Not reported	(i) Gastrostomy tube placement/feeding/nasogastric tube feeding (ii) Training to parents/caregivers (iii) Dietary modification	Weight Length/ Height TSF Mid- arm muscle area, WAZ, HAZ	Disaggregated data were not reported for children with CP. However, the results show that all patients benefited from enteral feeding with a statistically significant increase in weight ($p<0.01$). There was significant weight gain ($p<0.01$), however, no differences in z scores regarding height and weight/height was observed.	Complications of GTF, Mortality

References

1. Jadi, J.; Hyder, S.; Rodriguez Ormaza, N.P.; Twer, E.; Phillips, M.; Akinkuotu, A.; Reid, T.D. Evaluation of Complications and Weight Outcomes in Pediatric Cerebral Palsy Patients With Gastrostomy Tubes. *The American Surgeon* **2021**, 00031348211034753.
2. Haberfellner, H.; Schwartz, S.; Gisel, E.G. Feeding skills and growth after one year of intraoral appliance therapy in moderately dysphagic children with cerebral palsy. *Dysphagia* **2001**, *16*, 83-96.
3. VERNON-ROBERTS, A.; Wells, J.; Grant, H.; Alder, N.; Vadamalayan, B.; Eltumi, M.; Sullivan, P.B. Gastrostomy feeding in cerebral palsy: enough and no more. *Developmental Medicine & Child Neurology* **2010**, *52*, 1099-1105.
4. Sullivan, P.B.; Juszczak, E.; Bachlet, A.M.; Lambert, B.; Vernon-Roberts, A.; Grant, H.W.; Eltumi, M.; McLean, L.; Alder, N.; Thomas, A.G. Gastrostomy tube feeding in children with cerebral palsy: a prospective, longitudinal study. *Developmental medicine and child neurology* **2005**, *47*, 77-85.
5. Henderson, R.C.; Grossberg, R.I.; Matuszewski, J.; Menon, N.; Johnson, J.; Kecskemethy, H.H.; Vogel, L.; Ravas, R.; Wyatt, M.; Bachrach, S.J. Growth and nutritional status in residential center versus home-living children and adolescents with quadriplegic cerebral palsy. *The Journal of pediatrics* **2007**, *151*, 161-166.
6. Campanozzi, A.; Capano, G.; Miele, E.; Romano, A.; Scuccimarra, G.; Del Giudice, E.; Strisciuglio, C.; Militerni, R.; Staiano, A. Impact of malnutrition on gastrointestinal disorders and gross motor abilities in children with cerebral palsy. *Brain and Development* **2007**, *29*, 25-29.
7. Kernizan, D.; Mintz, D.; Colin, M.; Lee, M.; Yoakam, L.; Chen, Y.P.; Iofel, E.; Koniaris, S.; Weidner, M. Outcomes and Safety of Blenderized Tube Feedings in Pediatric Patients: A Single Center's Experience. *Journal of pediatric gastroenterology and nutrition* **2020**, *71*, e124-e128.
8. Dipasquale, V.; Catena, M.A.; Cardile, S.; Romano, C. Standard polymeric formula tube feeding in neurologically impaired children: a five-year retrospective study. *Nutrients* **2018**, *10*, 684.
9. Clawson, E.; Kuchinski, K.; Bach, R. Use of behavioral interventions and parent education to address feeding difficulties in young children with spastic diplegic cerebral palsy. *NeuroRehabilitation* **2007**, *22*, 397-406, doi:10.3233/NRE-2007-22506.
10. CORWIN, D.; ISAACS, J.; GEORGESON, K.; Bartolucci, A.A.; Cloud, H.H.; CRAIG, C.D. Weight and length increases in children after gastrostomy placement. *Journal of the American Dietetic Association* **1996**, *96*, 874-879.
11. Kong, C.-K.; Wong, H.-S.S. Weight-for-height values and limb anthropometric composition of tube-fed children with quadriplegic cerebral palsy. *Pediatrics* **2005**, *116*, e839-e845.
12. Rempel, G.R.; Colwell, S.O.; Nelson, R.P. Growth in children with cerebral palsy fed via gastrostomy. *Pediatrics* **1988**, *82*, 857-862.
13. Arrowsmith, F.; Allen, J.; Gaskin, K.; Somerville, H.; Clarke, S.; O'LOUGHLIN, E. The effect of gastrostomy tube feeding on body protein and bone mineralization in children with quadriplegic cerebral palsy. *Developmental Medicine & Child Neurology* **2010**, *52*, 1043-1047.
14. Mahant, S.; Friedman, J.N.; Connolly, B.; Goia, C.; Macarthur, C. Tube feeding and quality of life in children with severe neurological impairment. *Archives of disease in childhood* **2009**, *94*, 668-673.
15. Pinnington, L.; Hegarty, J. Effects of consistent food presentation on efficiency of eating and nutritive value of food consumed by children with severe neurological impairment. *Dysphagia* **1999**, *14*, 17-26.
16. Gisel, E.G.; Tessier, M.-J.; Lapierre, G.; Seidman, E.; Drouin, E.; Filion, G. Feeding management of children with severe cerebral palsy and eating impairment: an exploratory study. *Physical & occupational therapy in pediatrics* **2003**, *23*, 19-44.
17. Brant, C.Q.; Stanich, P.; Ferrari Jr, A.P. Improvement of children's nutritional status after enteral feeding by PEG: an interim report. *Gastrointestinal endoscopy* **1999**, *50*, 183-188.
